# Bionic Cooling Skin for Infected Wound Healing

**DOI:** 10.1007/s40820-026-02240-6

**Published:** 2026-05-28

**Authors:** Shuo Shi, Huiqun Zhou, Yang Ming, Xiong Zhou, Hanbai Wu, Haipeng Ren, Lung Chow, Jing Su, Daming Chen, Bin Fei, Joselito M. Razal, Xungai Wang

**Affiliations:** 1 Joint Research Centre for Fiber Innovations and Renewable Materials, School of Fashion and Textiles, The Hong Kong Polytechnic University, Hong Kong S.A.R, 999077 People’s Republic of China; 2 Department of Biomedical Engineering, City University of Hong Kong, Hong Kong S.A.R, 999077 People’s Republic of China; 3 College of Textile Science and Engineering, Jiangnan University, Wuxi, 214122 People’s Republic of China; 4 State Key Laboratory of Bio-Based Fiber Materials, College of Textile Science and Engineering, Zhejiang Sci-Tech University, Hangzhou, 310016 People’s Republic of China

**Keywords:** Nanofibers, Janus structure, Breathable, Bionic skin, Wound healing

## Abstract

Infected wounds can lead to delayed healing, suppuration, and potentially life-threatening complications, making their management critically important. An ideal wound dressing should possess key characteristics such as high protective function, comfortable user experience, and effective antibacterial efficiency. However, a single dressing that integrates all these functions is rarely achieved. Herein, we developed a “bionic cooling skin” for infected wound management based on hierarchical nanofiber construction. This was achieved by integrating solvent welding technology with single-sided metal–organic frameworks (MOFs) that generate visible light-responsive reactive oxygen species (ROS, band gap = 2.56 eV). The designed bionic skin promotes rapid healing of infected wounds, and the healing mechanism has been confirmed by gene analysis. This advanced dressing closely mimics natural skin, exhibiting similar mechanical properties (*σ*_max_ = 21.6 MPa; *ε*_max_ = 54%), and high air and moisture permeability (> 1.8 mL s^−1^ and > 12.5 kg m^−2^ d^−1^), respectively. Furthermore, the bionic cooling skin reduces the local temperatures of wounds exposed to sunlight by 4 °C, mitigating heat gain through high mid-infrared emissivity. This innovative bionic wound dressing not only enhances comfort and healing efficacy but also advances our understanding of wound repair mechanisms, holding significant promise for future wound care and biomedical material design.

**Supplementary Information:**

The online version contains supplementary material available at 10.1007/s40820-026-02240-6.

## Highlights

- A bionic cooling skin with a hierarchical Janus nanofiber structure was fabricated by combining solvent welding technology with single-sided visible light-responsive metal–organic frameworks.
- This unique design simultaneously realized effective passive cooling (~4 °C reduction under sunlight) through high mid-infrared emissivity and on-demand antibacterial activity via photocatalytic reactive oxygen species generation.
- The bionic skin dressing closely mimics natural skin in mechanical properties and permeability while demonstrating superior healing performance for infected wound, with mechanistic insights supported by comprehensive gene expression analysis.

**Supplementary Information:**

The online version contains supplementary material available at 10.1007/s40820-026-02240-6.

## Graphical abstract

**Figure:**
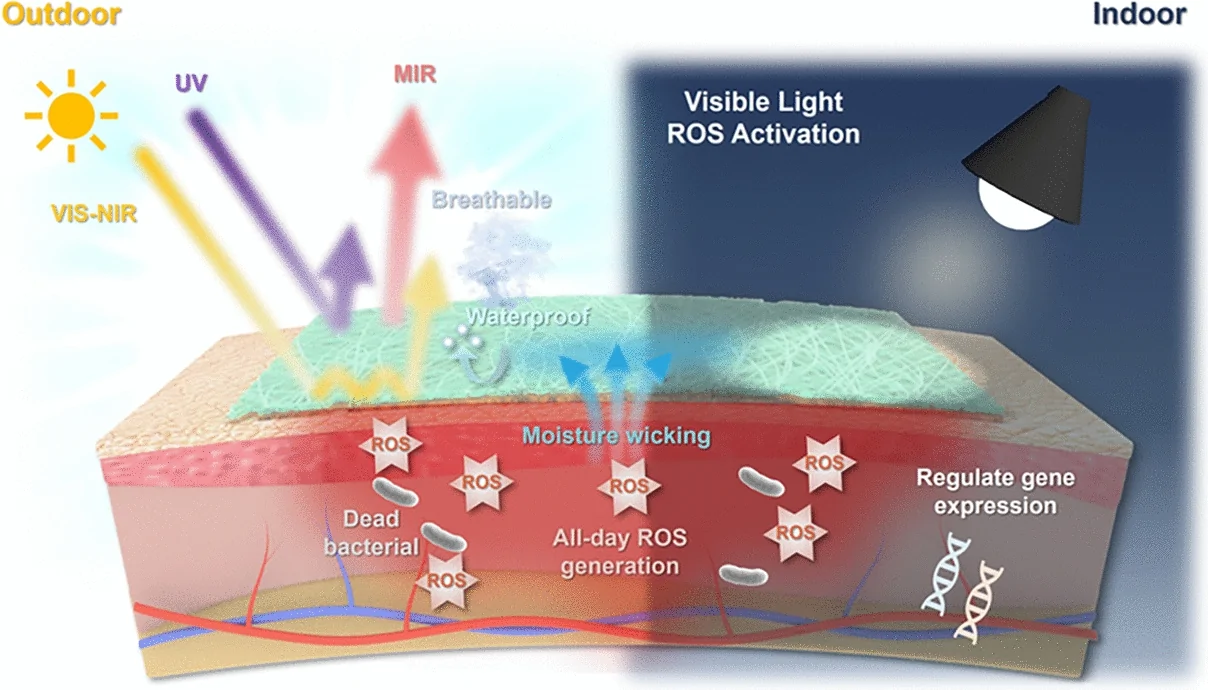

## Introduction

The skin, comprising the epidermis and dermis, is the largest organ of the human body, which serves as the primary defense mechanism. It effectively protects against mechanical damage, chemical exposure, microorganisms, and ultraviolet radiation [1–4]. In adults, the skin covers an area of approximately 1.5–2 square meters [5, 6]. Skin wounds represent a major health concern, with over 300 million surgeries performed globally each year, each resulting in surgical wounds [7]. Postoperative wound infections remain a frequent complication, affecting 5%–20% of surgical patients. Bacterial infection is a leading cause of delayed healing, chronicity, and increased morbidity across diverse wound types, including traumatic, surgical, and burn injuries. These infected skin wounds significantly impair patients’ quality of life and impose substantial economic and healthcare burdens.

Wound repair is a complex biological process influenced by numerous factors, with bacterial infection being a significant impediment. Infections may delay healing, exacerbate inflammation, and damage newly formed tissue [8–12]. Conversely, an adequate blood supply is crucial for delivering oxygen and nutrients, thereby facilitating cell proliferation and tissue repair, while insufficient blood supply can lead to tissue hypoxia and delayed healing [13]. A moderately moist environment also supports cell migration and tissue regeneration, promoting healing [14]. Traditional wound dressings have significantly contributed to wound management [15]. However, traditional dressings have limitations: gauze dressings may adhere to wounds, causing pain during changes; foam dressings are costly; and hydrocolloid dressings are unsuitable for infected wounds. Thus, substantial research potential remains in the development of optimal wound dressings, with particular focus on two key aspects: wear comfort and functional efficacy.

Current research on wound dressings has primarily focused on balancing comfort and functionality. For instance, nanofiber dressings, produced via electrospinning offer high surface area and breathability, with drugs and growth factors encapsulated in nanoparticles for controlled release [16–18]. Electrospinning technology provides advantages in preparing wound dressings, producing fiber structures akin to the extracellular matrix (ECM), which simulate cellular environments and regulate the cell microenvironment [19]. Research also indicates that piezoelectric materials, such as PVDF nanofibers, can enhance cell differentiation and proliferation [20]. By designing the diameter and structure of nanofibers, the microenvironment near the skin can be regulated, thereby improving comfort and managing the temperature and humidity of the wound area, which is beneficial to wound repair [6, 21]. Chitosan, known for its biocompatibility and antibacterial properties, is extensively studied for wound care, and the antibacterial properties of nanosilver have been explored to treat infected wounds [22, 23]. However, given the overuse of antibiotics, novel antibacterial materials are crucial for wound repair and combating resistant bacteria. Zeolitic imidazolate frameworks (ZIFs) represent a category of metal–organic frameworks (MOFs) that exhibit topological isomorphism with zeolites. ZIFs can be developed with a controllable structure, efficient reactive oxygen species (ROS) generation, and a simple preparation process to obtain effective antibacterial properties [24, 25]. Nevertheless, the design of visible light-responsive ZIF-based antibacterial materials remains a significant challenge. In summary, although substantial progress has been made in enhancing the comfort and functionality of wound dressings, there remains considerable scope for further research, particularly in the development of novel materials such as ZIFs that integrate comfort, high-efficiency antibacterial properties, and visible light responsiveness.

In this study, we developed a bionic cooling skin for wound dressing with a Janus structure by integrating functional ZIFs and hierarchical PVDF nanofibers. This bionic design mimics key physiological features of native skin, including favorable mechanical compatibility, breathability, and moisture permeability. By employing solvent welding, single-sided ZIF modification, visible light-responsive ROS functionality, passive cooling performance, and gene regulation, we effectively facilitated the rapid healing of infected wounds. Solvent welding technology enabled the formation of robust physical bonding points between electrospun nanofibers, imparting excellent mechanical properties to the fabricated membranes. Control over nanofiber welding and pore size ensured the air and moisture permeability of the bionic skin, closely mimicking human skin function. The Janus structure design provides passive cooling under sunlight and enhances fluid diffusion in the inner layer, thereby improving wearing comfort. ZIF modification endowed the dressing with visible light-responsive ROS antibacterial functionality, and RNA sequencing analysis demonstrated that the designed cooling skin effectively regulated gene expression for enhanced antibacterial property and wound healing. Overall, the structural and functional design of the Janus membrane improves dressing comfort and advances the understanding of wound repair mechanisms, thereby promoting wound management strategies and the development of novel materials.

## Materials and Experiment Section

### Materials

All materials were purchased and used without further purification. Polyvinylidene fluoride (PVDF) was provided by HK Keji Rabbit Biochemical Limited. Zinc nitrate hexahydrate (Zn(NO_3_)_2_·6H_2_O, ≥ 98.0%), 2-methylimidazole (2-MeIM, ≥ 97.0%), Dimethylformamide (DMF, ≥ 99.9%) were purchased from Alfa Aesar. LiCl (≥ 99.5%) and iron (II) chloride hexahydrate (FeCl_2_·6H_2_O, ≥ 99.0%) were purchased from Aladdin. Methanol and ethanol (ACS grade) were purchased from Anaqua Co Ltd.

### Experiment Section

#### Electrospinning of PVDF Membrane and Solvent Welding

PVDF electrospun membrane was fabricated by the following steps. 20% PVDF solution with 0.2% LiCl in DMF was prepared for electrospinning. The applied voltage of 18 kV, feed speed of 0.2 mL h^−1^, and the tip to collect of 15 cm were used for preparing the PVDF electrospun membrane via an electrospun machine supplied by Tongli Tech Co., Ltd in PolyU. Solvent welding means the mixed solution of DMF/H_2_O (v/v, 50/50) was used to weld nanofibers to enhance its mechanical properties [26]. Specifically, for a 10 cm × 10 cm electrospun PVDF membrane, 2 mL of mixed solvent (DMF/H₂O, v/v = 50/50) was uniformly cast onto the membrane surface. The membrane was then placed in an oven at 100 ℃ for 1h to complete the welding process.

#### Plasma Treatments on PVDF Membrane

The PVDF membrane was cut to the size of 10 cm × 10 cm and placed on a quartz glass. The membrane was then transferred into a plasma cleaner (Harrick Plasma, Ltd., USA) with a power of 38 W for 2 min under an air environment. For single-face plasma-treated PVDF, a PVA solution was initially applied to one side of the PVDF membrane, followed by plasma treatment.

#### Growth of ZIFs on PVDF Membrane

The plasma-treated membrane (10 cm × 10 cm) was then immersed into a mixture of Zn and Fe methanol solution with increasing iron concentration (*M*_total_ = 15 mmol, Zn: Fe = 100, 95, 90, 80, 70 in molar) in a beaker (BKMAMLAB Co. Ltd., China). Another solution (10 mL) prepared by dissolving 1.56 g 2-MeIM powder into 10 mL MeOH was slowly added into the above beaker. This device was then sealed by paraffin film and transferred on an orbital shaker for subsequent 8h ZIFs nucleation and growth. Finally, the ZIFs membrane was washed by deionized water, and ethanol to remove loosely grown ZIFs. The specific formula used in the experiment was given in Table S1, and the corresponding samples were named PVDF@FeX-ZIF8. For PVDF@FeX-ZIF8 (Janus) membranes, the same procedures were followed, with the only difference being the use of single-face plasma-treated PVDF.

#### Physical Characterizations

Scanning electron microscopy (SEM) images were obtained from Tescan MIRA 3, Czech (accelerating voltage = 20 kV). Transmission electron microscopy (TEM) was conducted on JEOL 2100F, Japan (operating voltage = 200 kV). X-ray diffraction (XRD) patterns were collected on a Rigaku SmartLab 9 kW-Advance, Japan, using monochromatic Cu kα radiation (*λ* = 0.154 nm) at a scan rate of 7° min^−1^. Raman spectroscopy was taken using a NomadicTM 3-in-1 microscope, America, with He–Ne laser excitation (*λ* = 532/785 nm). The reflectance (*R*(*λ*)) and transmittance (T(λ)) across 2.5–15 μm were measured by a Fourier Transform Infrared Spectrometer (FT-IR, Spectrum 100, PerkinElmer) with a diffuse gold integrating sphere. Electron paramagnetic resonance spectra (EPR) were measured on paramagnetic resonance spectroscopy, FA-200, JEOL at 300 K. A 300W Xe lamp (PLS-SXE300, Beijing Perfect Light Tec. Co., Ltd.) for stimulating sunlight was placed at a distance of 30 cm from the membrane. An infrared camera (FLIR E33) was utilized to take thermal images, and a thermocouple was used to detect surface temperature. The water contact angle (WCA) was measured using an SDC-350 contact angle measurement equipment (Dingsheng, Ltd., China). The air permeability of the designed wound dressings was evaluated by an SDL air permeability instrument. The water vapor transmission rate (WVTR) of the wound dressings was measured by the constant temperature and humidity chamber. The equation for calculating WVTR (kg m^−2^ d^−1^) was given in Eq. (1).

1 $$\mathrm{W}\mathrm{V}\mathrm{T}\mathrm{R}=({M}_{1}-{M}_{2})/(S\cdot t)$$

in which *M*_1_ is the initial weight of the test jar; *M*_2_ (g) is the final weight of the test jar; *t* (h) is the testing time; and *S* (m^2^) is the area of measurement of WVTR.

UV–Vis diffuse reflection spectrum (UV–Vis DRS) was obtained by UV Cary-300, Shimadzu, UK equipped with an integrating sphere using white BaSO_4_ as reference. XPS and UPS were conducted on Thermo Scientific Nexsa (UK), using a monochromatic and focused Al Kα source (1486.6 eV) and He I source (21.2 eV). The band gap, valence band and conduction band of the prepared catalysts can be calculated according to Eqs. (2–4) based on UPS and VB-XPS results:

2 $${E}_{g}={\mathrm{H}\mathrm{O}\mathrm{M}\mathrm{O}}_{\mathrm{p}\mathrm{o}\mathrm{t}\mathrm{e}\mathrm{n}\mathrm{t}\mathrm{i}\mathrm{a}\mathrm{l}}-{\mathrm{L}\mathrm{U}\mathrm{M}\mathrm{O}}_{\mathrm{p}\mathrm{o}\mathrm{t}\mathrm{e}\mathrm{n}\mathrm{t}\mathrm{i}\mathrm{a}\mathrm{l}}$$

3 $$\phi ={21.22eV- E}_{\mathrm{c}\mathrm{u}\mathrm{t}-\mathrm{o}\mathrm{f}\mathrm{f}}$$

4 $${\mathrm{H}\mathrm{O}\mathrm{M}\mathrm{O}}_{\mathrm{E}\mathrm{v}\mathrm{a}\mathrm{c}}= \phi +{\mathrm{H}\mathrm{O}\mathrm{M}\mathrm{O}}_{E \mathrm{f}\mathrm{e}\mathrm{r}\mathrm{m}\mathrm{i}}$$

#### Photoelectrochemical Measurements

The obtained photo/electrocatalysts (5 mg) were dispersed into a mixture of iso-propanol (200 μL); ethanol (200 μL) and DI H_2_O (580 μL, conductivity = 0.26 μS cm^−1^) with an extra addition of Nafion dispersion (20 μL, 5 wt%). After achieving uniform dispersion of catalyst in an ice bath under sonication for 1h, 10 μL of dispersion was drop-casted onto one side of 1 cm^2^ FTO glass (*A*_geometric_ = 1 cm^2^). In all cases, FTO glasses were washed with acetone, ethanol, and DI H_2_O under sonication several times. The casted electrode was dried under infrared irradiation for 30 min to yield a mass load of ~ 0.2 mg cm^−2^.

The electrochemical measurements were taken at the electrochemical workstation (CHI760E, Shanghai Chenhua Science Technology Corp., Ltd.). A typical three-electrode configuration including Pt plate (2 cm × 1 cm, 0.1 mm) as a counter electrode; Ag/AgCl (saturated KCl solution) as reference electrode, fluorine-doped tin oxide conductive glass (FTO, cut with 1 cm × 1 cm). N_2_ saturated 0.1 M Na_2_SO_4_ (pH = 7.1) functioned as an electrolyte. Chopped light current–time (i–t) curves were obtained at open-circuit potential (OCP) versus Ag/AgCl reference electrode with 30-s light on and off intervals.

#### Proliferation and Cytotoxicity Test of Wound Dressing

Fibroblasts NIH3T3 were used as standard cells to test the proliferation and toxicity of the material. Cells were cultured with complete medium (Dulbecco’s modified Eagle medium, 10% fetal blood serum, and 1% penicillin–streptomycin) in a 37 °C incubator. The CCK-8 assay and Live/Dead staining kits were used to detect cells at the target time points [27]. Cell proliferation was analyzed using a microplate reader (SpectraMax), while cell toxicity was assessed through cell images captured by fluorescence microscopes (Nikon TS-100F).

#### Infected Wound Healing Study and Analysis

In this study, six-week-old male Kunming mice were used for in vivo experiments, all animal experiments followed the guidelines of animal ethics (Shenzhen TOP Biotechnology Co., Ltd., approval number: TOP-IACUC-2024-0090). The mice were anesthetized with isoflurane, and a 10 mm diameter section of dorsal skin was excised. The wound was then infected with 1 × 10^7^ CFU mL^−1^ of *Staphylococcus aureus*. We established a negative control group C (−) with untreated wounds and a positive control group C (+) with antibiotic-treated wounds. Throughout the experiment, all mice were exposed to white light until the wounds were nearly healed. Weight measurements and images were taken at specified intervals to monitor wound healing progress. At the end of the experiment, blood, dorsal skin, and major organs were collected for further analysis. Blood samples underwent routine examination, and major organs were assessed for material toxicity using H&E staining. Skin tissues were analyzed by H&E, Masson, and immunohistochemical (IHC) staining to evaluate healing in different treatment groups. For IHC staining, we selected angiogenesis markers CD31 and CD34, the inflammatory factor TNF-α, and the antifibrotic factor TGF-β1 for verification. Tissues for RNA sequencing were pre-cooled, and sequencing was conducted.

Majorbio Bio-Pharmaceutical Technology Co., Ltd. on Illumina NovaSeq/Hiseq Xten (Illumina).

#### Mechanical Modeling for Random and Bonded Nanofiber Mat

For unbound fiber mats, use a Python script to generate random fibers within a cubic area with a length of 30, a width of 10, and a height of 5. The nanofiber content in space is 90%. Random fiber generation was performed using the RSE algorithm proposed by Yang et al. [28]. This algorithm ensures that the fibers are not in contact with each other. Therefore, the orientation of the nanofibre mats is determined by adjusting the length of the fibers. In other words, when the length of the nanofibres is equal to the length of the cube space, the algorithm aligns most of the fibers along the length of the cube space to ensure that the fibers do not touch each other. The same rules were used for angles such as 0°, 30°, 45°, and 60°. For the bonded nanofiber mat, since the contact points are bonded, the nanofiber mats at different angles can be regarded as having the same dense three-dimensional network structure. All nanofibers are considered as beam elements with circular cross sections. For nanofibers without bonding, most of the macroscopic tensile strength of the nanofiber mat is provided by microscopic friction. When the friction reaches the extreme value, the nanofiber mat will break. For bonded fibers, the macroscopic tensile capacity of the fiber mat is determined by friction, the strength of the bonding point, and the strength of the nanofiber itself. Therefore, it is not only necessary to set the friction behavior but also to give the fiber and bonding point fracture properties.

#### RT-PCR (Quantitative Reverse Transcription Polymerase Chain Reaction) for Angiogenesis and Anti-inflammation Gene Determination

The total RNA of cells co-cultured with membranes was extracted using the TRIZOL reagent (Invitrogen, USA). RNA was measured using a Nanodrop to detect concentration. An equivalent amount of RNA was transcribed into complementary DNA (cDNA) according to the instructions of the Reverse Transcription Kit (Invitrogen, USA). Total cDNA samples were subsequently stored at − 20 °C. Then, the qPCR for angiogenesis and anti-inflammation genes was performed using SYBR Green and a Real-Time PCR Detection System (BIOER, China). Quantification of target genes was evaluated by 2-∆∆CT method. The ΔCt value of each sample was calculated by comparing it with the Ct values of a housekeeping gene, beta-actin. Details of primers are shown in Table S2.

#### Theoretical Simulation of Band Gap

The density functional theory (DFT) simulations were carried out with CASTEP module. The Perdew–Burke–Ernzerhof (PBE) functional, within the generalized gradient approximation (GGA), was chosen to characterize the electron–electron interaction. The projector augmented-wave pseudopotential (PAW) was applied with a kinetic energy cutoff of 520 eV, which was utilized to describe the expansion of the electronic eigenfunctions. All atomic positions were fully relaxed until energy and force reached a tolerance of 1 × 10^−6^ eV and 0.02 eV Å^−1^, respectively. A Gamma 3 × 3 × 3 k point was employed for density of states calculations.

## Results and Discussion

### Characteristics of PVDF@FeX-ZIF8 Electrospun Membrane

**Fig. 1:**
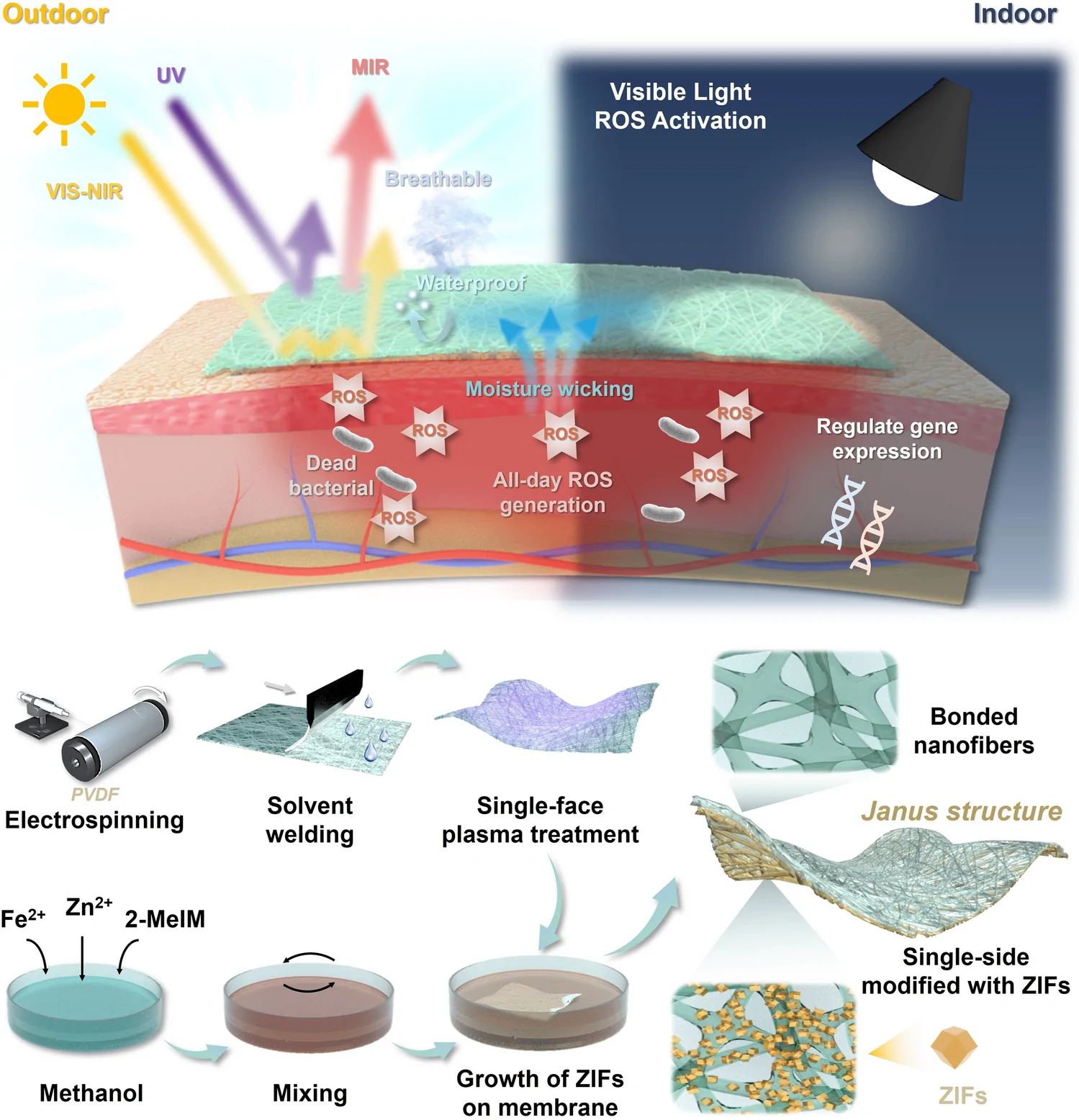
Schematic diagram of the bionic cooling skin design: a Janus structure, good breathability, antibacterial property, and passive cooling performance for accelerating infected wound healing

An ideal wound dressing should provide comfort and facilitate wound repair. To enhance comfort, we employed solvent welding technology for in situ welding of the electrospun membrane, significantly improving its mechanical properties to closely resemble those of natural skin, while also ensuring breathability and waterproofing. The Janus structure of the membrane features single-sided Fe-modified ZIF8, which not only offers effective thermal management by reducing local high temperatures from sunlight exposure but also provides good intermediate infrared emissivity for passive cooling. The nanofibers exhibit an ECM-like structure that supports wound healing, and annealing technology enhances the β phase content, promoting cell differentiation and accelerating healing. Additionally, the Fe-modified ZIF8 membrane demonstrates excellent antibacterial properties, effectively aiding the healing of infected wounds and regulating wound healing-related gene expression. The design of the PVDF@ZIF8 bionic skin is depicted in Fig. 1.

**Fig. 2:**
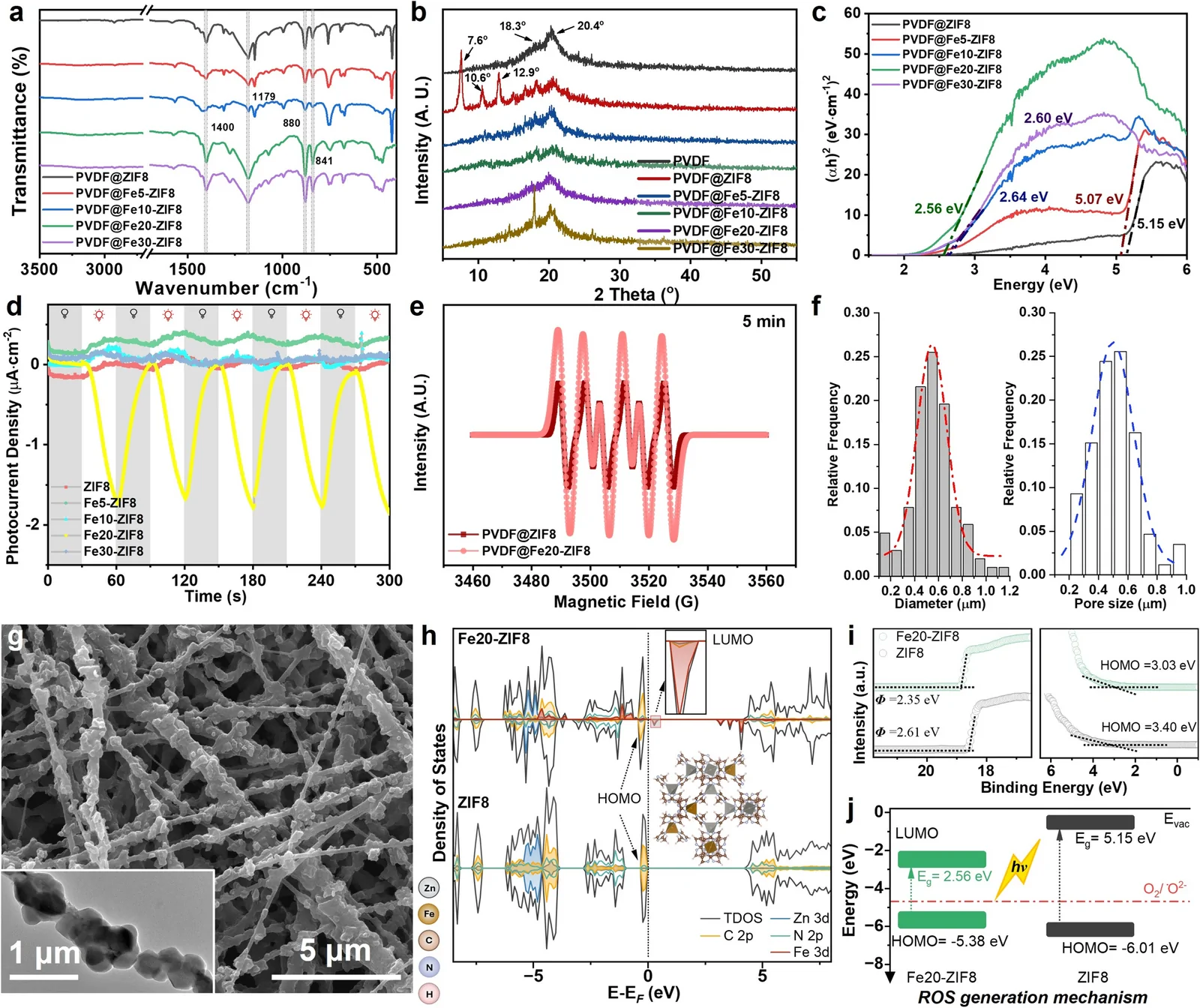
Structure and characteristics of PVDF@FeX-ZIF8 bionic skin. **a–c** FT‐IR spectra, XRD pattern, and Tauc’s plots of PVDF@FeX-ZIF8 bionic skin. **d** Photocurrent generated by ZIF8 and Fe-dopped ZIF8. **e** ESR spectra of PVDF@FeX-ZIF8 bionic skin. **f** Distribution of the diameter of ZIF-loaded PVDF nanofibers and their pore size. **g** SEM and TEM images of PVDF@FeX-ZIF8 bionic skin. **h** Projected density of states (PDOS) of Fe20-ZIF8 and ZIF8 with labeled HOMO and LUMO. **i** UPS survey and valence band-XPS of Fe20ZIF8 and ZIF8 and **j** corresponding HOMO and LUMO levels (down) relative to O_2_/O_2−_ potential (− 4.68 eV vs. Evac)

The composition, structure, and performance of materials are the basic triangular relationship in materials science. In this section, the structure of iron-doped ZIF8 and the morphological characterization of iron-modified ZIF8 loaded on PVDF nanomembrane were confirmed and investigated. First, we confirmed the basic structure of PVDF. The peaks at 1179, 880, and 841 cm^−1^ were assigned to the C–F stretching, C–H wagging, and C–F bending, respectively in the PVDF macromolecule chain; the peak at 1400 cm^−1^ was assigned to C–H deformation (Figs. 2a and **S1**) [29]. The crystallization peaks at 18.3° and 20.3° are typical *α*-phase and *β*-phase PVDF, respectively (Fig. 2b) [30]. According to our previous report, annealing increases the content of *β*-phase in PVDF [20]. In this study, the annealing process was used to treat PVDF, which assists cell differentiation in tissue engineering applications. In addition, further XRD patterns suggest that the introduction of iron significantly decreases the crystallinity of ZIF8. As depicted in Fig. 2b, the dominant peaks at 7.6°, 10.6°, and 12.9° correspond to the (110), (200), and (211) planes of the ZIF8 crystal (JCPDS 00-062-1030), respectively [31]. With increasing doping level of iron atoms, the crystallinity of the frameworks decreases significantly (Fig. S2 and Table S3). Furthermore, we also analyzed the microstructure of the prepared Fe20-ZIF8-loaded PVDF membrane. Its fiber diameter and porosity distribution exhibit characteristics of a Gaussian distribution. The Fe20-ZIF8-loaded PVDF membrane possesses an average fiber diameter of ~ 0.55 μm, and the pore size is ~ 0.50 μm, while the pure PVDF membrane has an average fiber diameter of ~ 0.18 μm (Figs. 2f and S4). In addition, Fe20-ZIF8 shows good adhesiveness on PVDF nanofibers (Fig. 2g). Through elemental analysis (Figs. 2g and S3), we further confirmed the composition, content, and distribution of each element, C, N, F, O, Fe, and Zn in the Fe20-ZIF8@PVDF fiber.

Meanwhile, the bandgap width of ZIF8 (Fig. 2c) was effectively adjusted by Fe doping. UV–Vis spectroscopy was conducted to investigate the bandgap tunability of ZIFs through Fe doping. As indicated in Figs. 2c and S5, an apparent absorption edge at ~ 240 nm can be observed in the ZIF 8-loaded PVDF membrane, suggesting a bandgap of 5.15 eV, which is comparable to reported values [32]. This bandgap is further narrowed to 2.56 eV due to the increasing doping level of Fe atoms in the ZIFs frameworks. Specifically, the iron atoms substitute for the Zn atoms by forming coordination bonds with 2-MeIM ligand for simulation simplicity. As shown in Fig. 2h, Zn atoms are substituted by Fe atoms to mimic the Fe-doped ZIF8 structure. The PDOS of Fe20-ZIF8 reveals the additional electronic states originating from the Fe 3*d* electrons [33, 34]. This significantly reduces the band gap between the highest occupied (HOMO) and the lowest unoccupied molecular orbitals (LUMO) with a gap value of ~ 2.5 eV. Furthermore, with the proposed configuration of Fe-N4 structure, the enlarged LUMO (Fig. 2h) illustrates the effect on the electronic structure of the *sp*2C atom of the organic linkers. Therefore, it is convinced that the iron atom is successfully coordinated with N atoms in the imidazole frameworks. Such coordination configuration denotes “mid-gaps” between HOMO and LUMO, which significantly affects the light absorption behavior of candidates. Moreover, the ultraviolet photoelectron spectroscopy (UPS) was conducted to determine the HOMO and LOMO levels of candidates. As depicted in Fig. 2i, the determined working function for Fe20-ZIF8 and ZIF8 is 2.35 and 2.61 eV, respectively, contributing a distinctive molecular orbital level (Fig. 2j). The reduced gap in Fe20-ZIF8 can effectively trigger the O_2_/O_2_^−^ redox reaction (− 4.86 eV vs. Evac) upon visible light absorption. Therefore, the excitation wavelength is effectively adjusted from the UV band to the visible light band by Fe doping. The transient photocurrent of FeX-ZIF8 was recorded at open-circuit potential upon visible light irradiation (*λ* > 420 nm) with a concentration of 1 kW m^−2^. This photocurrent measurement aims to reveal the capability of ZIFs to produce free electrons after light stimulation. As depicted in Fig. 2d, the original ZIFs exhibit anodic current, suggesting the characteristics of n-type semiconductors [35]. Due to the large band gap of ZIF8 (5.15 eV) and low visible light absorption capacity, there is limited collected photocurrent. After introducing iron ions to 20 at%, the photocurrent rises to a repeatable value of − 1.75 μA cm^−2^, demonstrating an optimal doping concentration. Moreover, increasing the doping concentration significantly influences the crystal structure of ZIFs (e.g., HOMO and LUMO orbitals), which leads to insufficient charge transfer and results in no detectable photocurrent in Fe30-ZIF8. Subsequently, electron paramagnetic resonance (EPR) analysis, directly using the Fe_x_-ZIF8-loaded membrane, was conducted to explore the electron transfer pathway of visible light-induced charges. Under these circumstances, 5,5-diemthyl-1-pyrroline N-oxide (DMPO) was employed as the spin-trapping agent to detect the presence of superoxide radicals (O_2_^−^), commonly regarded as reactive oxygen species (ROS) for immunotherapy [36, 37]. As shown in Fig. 2e, typical DMPO-·O_2_^−^ adducts signals (six characteristic peaks) are observed after 5 min of visible light irradiation (*λ* > 420 nm). This indicates the potential reduction of absorbed O_2_ by receiving free electrons (O_2_ + e^−^ → ·O_2_^−^). Compared with the original PVDF@ZIF8, about twice the signal magnitude can be observed, proving the efficient accumulation of ROS in PVDF@Fe20-ZIF8. Such a trend facilitates further improved inflammatory repairing capability of the membrane. In this situation, we confirmed that the reduced O_2_ acts as the ROS rather than Fe^2+^ or Zn^2+^ ions, which function as the active species responsible for bacterial inactivation.

### Mechanical Properties and Comfort of Bionic Janus-Structured Skin

The basic design principles of wound dressings include excellent mechanical properties, air permeability, and moisture permeability. Mechanical properties are fundamental to materials and significantly impact their application. Electrospun membranes typically have poor strength, posing a risk of rupture during use. As a wound dressing, such rupture may lead to secondary infections and severely affect healing efficiency. In designing these bionic Janus-structured wound dressings, we addressed the issue of poor strength through solvent welding, thereby achieving excellent moisture permeability, air permeability, and waterproof properties in the bionic skin wound dressing.

**Fig. 3:**
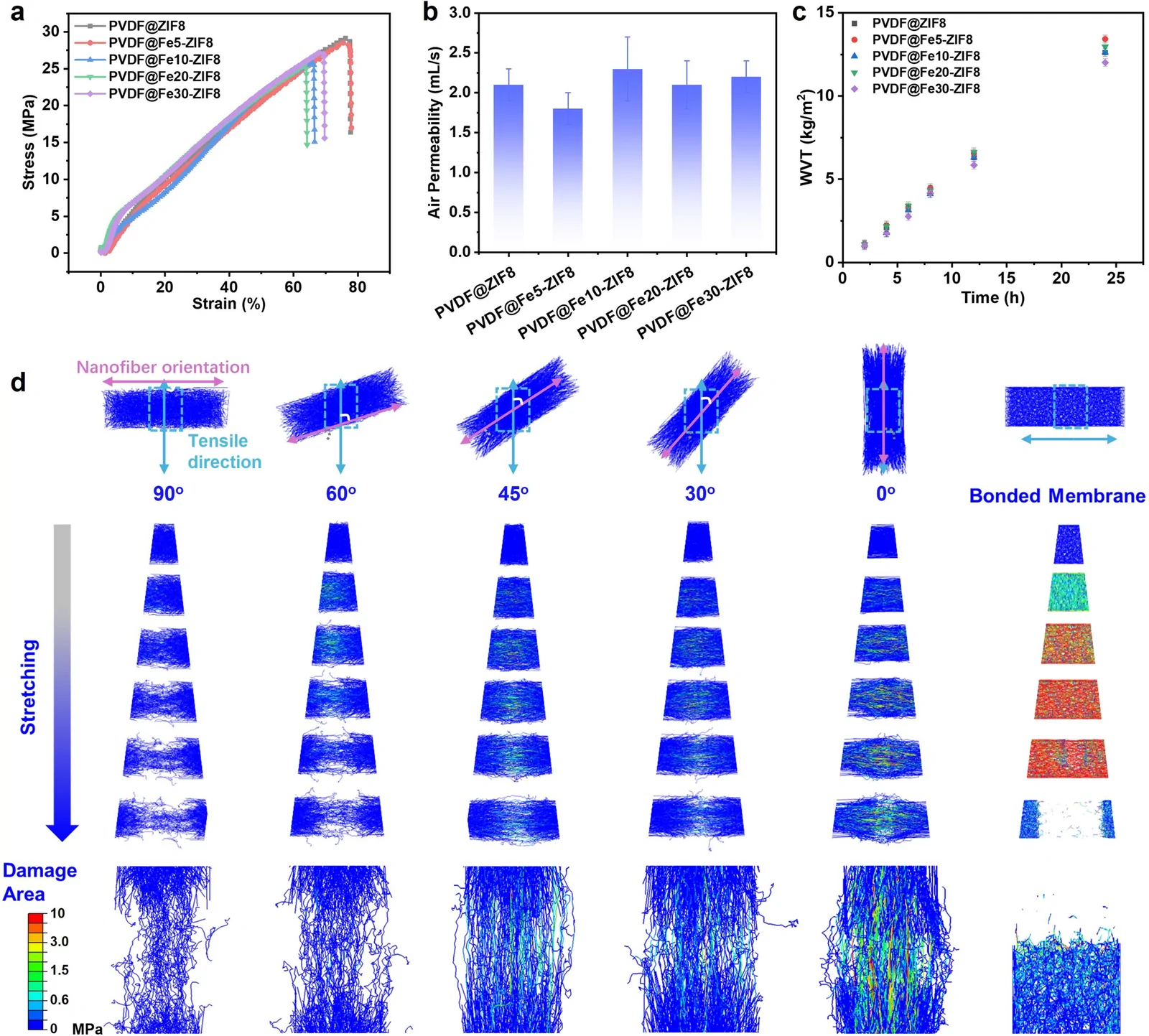
Mechanical property and breathability of PVDF@FeX-ZIF8 bionic skin. **a–c** Strain–stress curves, air permeability, and water vapor transmission property of PVDF@FeX-ZIF8 bionic skin. **d** Fracture processes of nanofiber membrane via modeling analysis before and after welded treatment

Compared with the mechanical strength of the original PVDF nanomembrane, which is about 12 MPa (Fig. S6), the strength of the PVDF nanomembrane loaded with ZIF8 can be effectively increased to more than 25 MPa through a solvent welding strategy (Fig. 3a). The resulting membrane exhibits strength similar to that of human skin, with a tensile strength of ~ 21.6 MPa and a failure strain of ~ 54% [38]. Additionally, the membrane demonstrates excellent particle protection (Fig. S7), with a filtration efficiency exceeding 99.8%. From a mechanical standpoint, the designed bionic wound dressing exhibits characteristics similar to real skin. Its air permeability and moisture permeability (Fig. 3b, c**)** exceed 1.8 mL s^−1^ and 12.5 kg m^−2^ d^−1^, respectively. Further, mechanical simulation results under the same tensile strain are presented in Fig. 3d, demonstrating that internal stresses in the highly oriented nanofiber mats increase as the angle *θ* between the tensile direction and the primary fiber alignment direction decreases. This indicates that the effective elastic modulus decreases with increasing orientation angle *θ*, leading to reduced resistance to deformation when fibers are more misaligned with the loading direction. For the bonded nanofiber mats, the stress distribution maps show a relatively uniform and high internal stress field. Moreover, the smooth fracture interfaces observed in the bonded mats suggest uniform distribution of internal forces throughout the structure. Compared to previous reports, this innovative bonding method avoids complex chemical modifications and high-temperature processing, enabling energy-efficient, large-scale production.

**Fig. 4:**
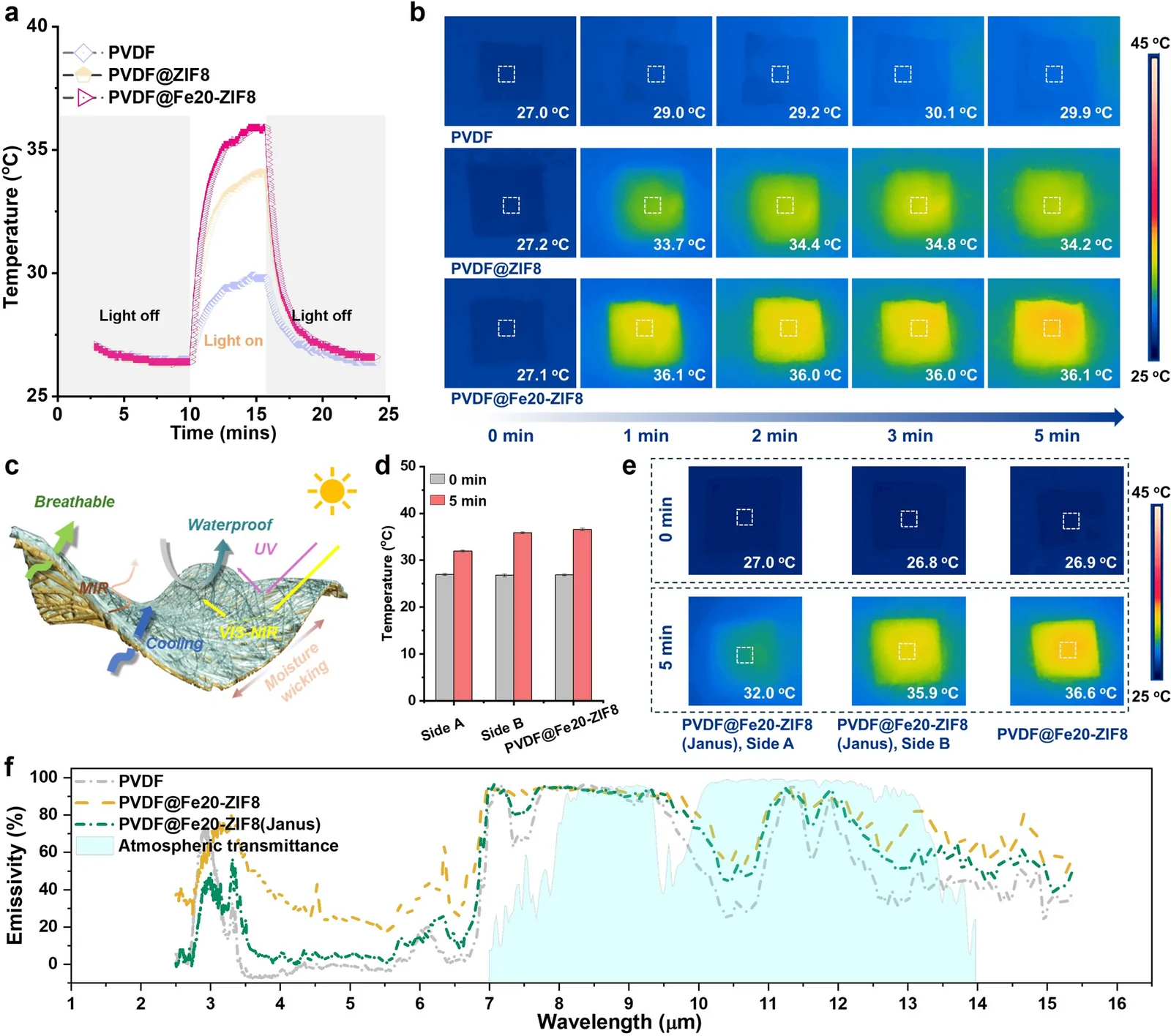
Photothermal property and passive cooling performance of bionic Janus-structured wound dressings. **a, b** Surface temperature response capacity of PVDF, PVDF@ZIF8, and PVDF@Fe20-ZIF8 under 1 sun illumination. **c** Janus structure design of PVDF@FeX-ZIF8 bionic skin with comfortability, meaning breathability, waterproofness, passive cooling performance, moisture wicking property, etc. **d, e** Comparison of the surface temperature regard to PVDF@Fe20-ZIF8, and bionic Janus-structured wound dressings under 1 sun illumination. **f** Emissivity spectra of PVDF, PVDF@ZIF8 (Janus), PVDF@Fe20-ZIF8 (Janus)

Compared to traditional electrospun fiber membranes, the designed wound dressing features a Janus structure. The inner layer is embedded with Fe20-ZIF8 nanoparticles to provide antibacterial properties, effectively aiding the healing of infected wounds. The outer layer, made of PVDF fibers, offers excellent sunlight reflection and mid-infrared transmission, enabling effective thermal management for comfortable wear in sunlight. We first compared the photothermal response performance of PVDF@Fe20-ZIF8 with PVDF@ZIF8 and PVDF. Compared with ZIF8@PVDF, PVDF@Fe20-ZIF8 has better photothermal performance reaching 36.1 °C in 5 min of simulated sunlight irradiation (Fig. 4a, b), which is 9 °C higher than the ambient temperature. For bionic Janus-structured wound dressings (PVDF@Fe20-ZIF8 (Janus)), compared with the PVDF@ZIF8 and PVDF@Fe20-ZIF8 membranes, the surface temperature was effectively reduced by 4 °C when Side A was on the outside (Fig. 4c–e). Meanwhile, we also conducted in vivo cooling experiments on a full-thickness excisional wound model in rats under realistic outdoor conditions (ambient temperature: 20–25 °C; solar irradiance: 115–195 W m^−2^, Shenzhen, China). Surface temperatures were simultaneously monitored using infrared thermal imaging. As shown in Fig. S8, the Janus PVDF@Fe20-ZIF8 dressing achieved a significant temperature reduction compared to controls. Relative to the uncovered wound, Janus PVDF@Fe20-ZIF8 dressing provides an average cooling of 1.7 °C. Therefore, the PVDF@Fe20-ZIF8 (Janus) can provide better comfort performance. Additionally, the prepared bionic wound dressing, PVDF@Fe20-ZIF8 (Janus), exhibits a high infrared emissivity (IE) (Figs. 4f and S9) in the atmospheric window of 7–14 μm, with an average IE of up to 80.7%. This is attributed to the presence of abundant IR-active chemical bonds such as C–C, C–O, and metal–O in the designed bionic wound dressing [39–41]. The hydrophobic and hydrophilic properties of the designed bionic wound dressing were evaluated (Fig. S10). The outer layer exhibits excellent hydrophobicity (WCA = 137°), while the inner layer is hydrophilic (WCA = 72°), facilitating the diffusion of exudate and providing wound protection. Therefore, during wear, the efficient transmission of mid-infrared radiation in the human body can be effectively achieved, providing the possibility of passive cooling and greatly improving the comfort of wound management [21].

### Evaluation of In Vivo Wound Healing and Biological Gene Expression

We quantitatively evaluated the wound healing process and systematically analyzed the in vivo antimicrobial efficacy, biocompatibility, and gene expression associated with the wound dressings during healing. With their comfortable design and effective antibacterial function under white light, these dressings facilitated successful wound healing.

**Fig. 5:**
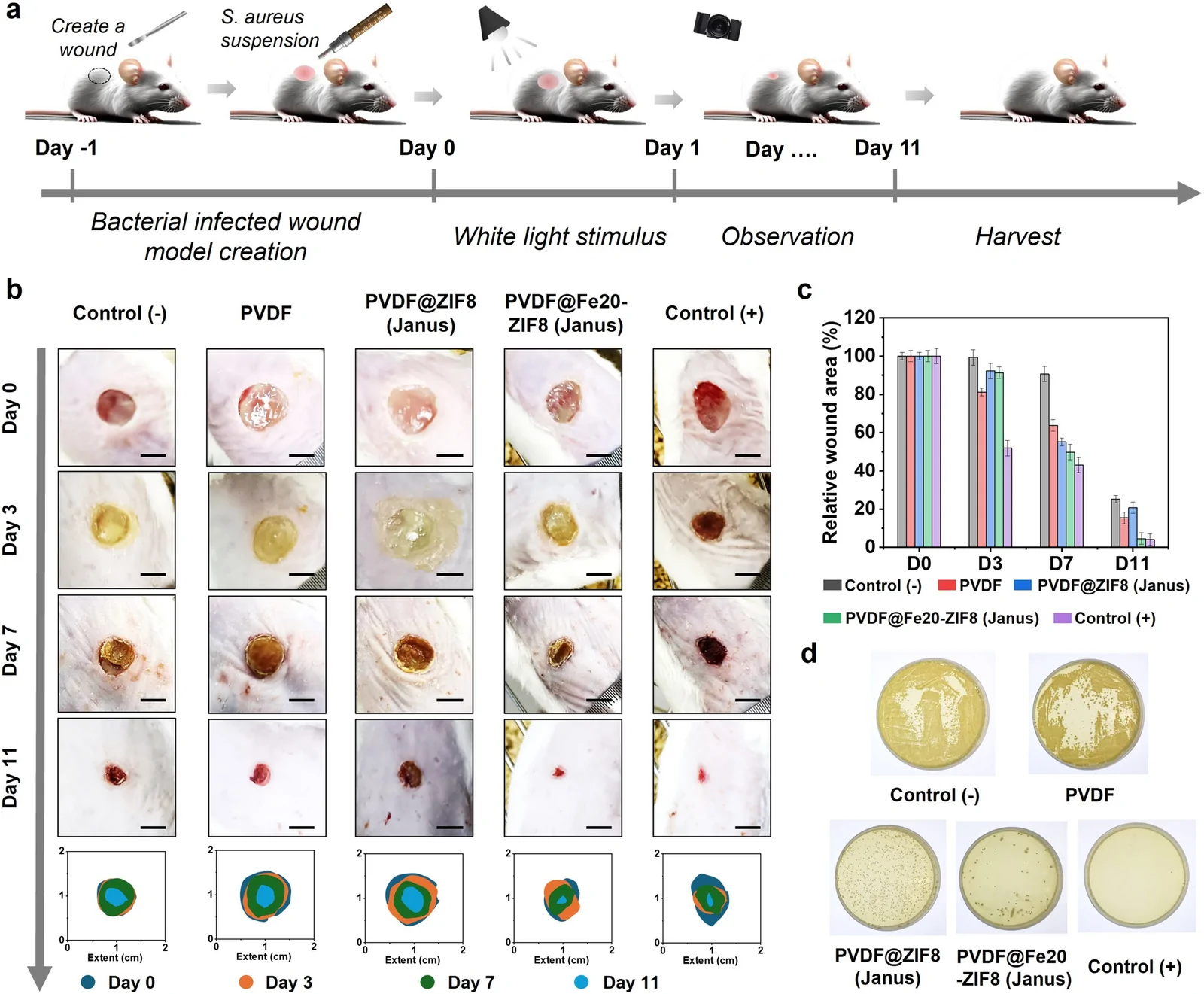
Investigation of the wound healing process for S. *aureus* infection wounds by Janus-structured wound dressings. **a** Schematic diagram of wound modeling and treatment processes. **b** Wound healing process and area maps with different wound dressings, PVDF, PVDF@ZIF8 (Janus), PVDF@Fe20-ZIF8 (Janus), and control groups over 11 days (scale bar 0.5 cm). **c** Quantitative analysis of wound healing area of mice dorsal skin. **d** In vivo antibacterial test

First, we established an infected wound on mice skin by *Staphylococcus aureus* and covered the wounds with different wound dressings, PVDF, PVDF@ZIF8 (Janus), and PVDF@Fe20-ZIF8 (Janus), under white light illumination. The negative control group (control-) was not treated, while the positive control group (control +) was treated with an amoxicillin aqueous solution. The animal experimental procedures are shown in Fig. 5a. The weight of mice in different groups showed a gradual increasing trend during the wound healing process (Fig. S11). In the early stages of wound infection, dressings without antibacterial function could not kill bacteria, so the wounds showed signs of redness, swelling, and inflammation. Meanwhile, the PVDF@ZIF8 (Janus) dressing-treated group and the positive control group showed far fewer adverse symptoms. After 11 days of treatment, the PVDF@Fe20-ZIF8 (Janus) dressing group and the positive control group had almost completely healed wounds compared to the other groups (Fig. 5b). This therapeutic outcome is likely attributed to the synergy between passive cooling and the Janus dressing’s antibacterial properties, the mechanism has been discussed in the following sections. Routine blood tests for all mice were within the normal range, and no abnormal inflammation response or immune responses were observed after treatment (Figs. S12–S14). By quantitative assessment of the wound healing (Fig. 5c), the PVDF@Fe20-ZIF8 (Janus) dressing and the positive control group exhibited more than twice the healing rate of the other groups. The in vivo antibacterial test showed that the antibacterial rate of the PVDF@Fe20-ZIF8 (Janus) wound dressing (Figs. 5d and S15) was approximately 97.1%, while that of PVDF@ZIF8 was about 43.2%. In vitro antibacterial assays confirmed the functional synergy within the Janus structure (Fig. S16). In the dark, pristine PVDF and PVDF@ZIF 8 showed negligible activity, while PVDF@Fe20-ZIF8 exhibited moderate intrinsic inhibition. This intrinsic activity under dark conditions could be potentially linked to the inherent antibacterial properties of the ZIF-based framework, such as the possible release of metal ions or localized surface interactions with bacterial membranes. Under visible light, PVDF@Fe20-ZIF8 achieved nearly 100% efficiency against both strains, suggesting that light-triggered ROS generation significantly contributes to the overall antibacterial performance. This possible dual-action mechanism combining intrinsic inhibition with photocatalytic activity may facilitate robust antimicrobial efficacy. Furthermore, the porous architecture of the top PVDF layer appears to allow sufficient visible light penetration to activate the underlying Fe-ZIF8, indicating that the Janus design effectively integrates thermal regulation and disinfection without compromising individual functional performance. Therefore, PVDF@Fe20-ZIF8 (Janus) can accelerate wound healing and provide antimicrobial properties.

**Fig. 6:**
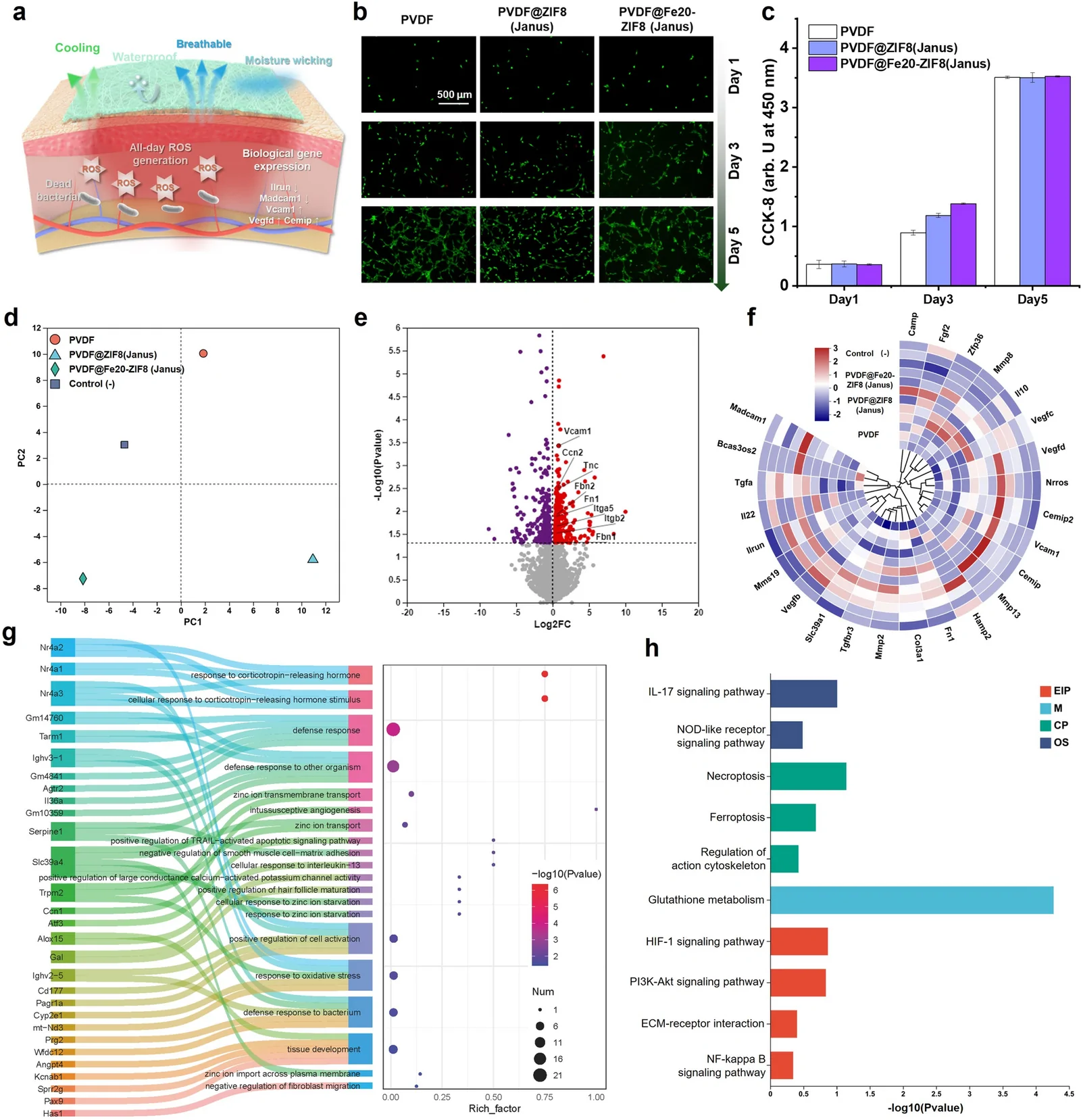
Gene expression during wound repair with bionic Janus-structured dressings. **a** Schematic diagram for wound healing process by bionic Janus-structured wound dressings. **b** Fluorescence staining of NIH3T3 co-cultured with wound dressings, PVDF@ZIF8 (Janus) and PVDF@Fe20-ZIF8 (Janus). **c** Proliferation of the fibroblasts incubated in conditional media with different wound dressings. **d** PCA of control group and wound dressings healed groups. **e** Volcano plot of significant genes expressed in PVDF@Fe20-ZIF8 (Janus) group. **f** Heatmap of differentially expressed genes related to wound healing. **g** Sankey diagram of genes related biological behaviors and their rich factor in PVDF@Fe20-ZIF8 (Janus) group. **h** KEGG enrichment analysis in PVDF@Fe20-ZIF8 (Janus) group compared with untreated group

To evaluate the biocompatibility of wound dressings, fibroblasts were co-cultured with the dressings, and cell proliferation and cytotoxicity over five days were investigated (Fig. 6b, c). During continuous co-culture of cells and materials for five days, cells grew over time without significant cell death, indicating the excellent biocompatibility of the applied wound dressings for infected wound management. To elucidate the potential antibacterial wound healing mechanisms, we further performed RNA sequence analysis of the collected skin tissues. Principal component analysis (PCA) of the samples confirmed significant separation between each group (Fig. 6d), and the Venn diagram (Fig. S17) illustrated the differentially expressed genes among groups. According to the differential gene statistics (Fig. S18), the PVDF@Fe20-ZIF8 (Janus) group had 312 upregulated genes and 301 downregulated genes compared to the control (-) group. Some genes positively associated with wound healing, such as integrins and fibronectin, were highlighted in the volcano plot (Fig. 6e). The PVDF@Fe20-ZIF8 (Janus) wound dressing eradicated bacteria and promoted the healing of infected wounds by regulating the expression of a series of key genes (Fig. 6f). The zinc and iron ions in the wound dressing upregulated numerous zinc finger proteins and iron-regulated transport proteins, which play vital roles in cell proliferation, differentiation, and immune responses. Compared with the control (-) group, PVDF@Fe20-ZIF8 (Janus) wound dressing group indicated downregulation of inflammation-related genes (Ilrun and Madcam1) [42, 43], upregulation of genes promoting angiogenesis (Vcam1, Vegfd, Vegfb, and Vegfc) [44], and upregulation of genes facilitating cell migration (Cemip, Cemip2, and Bcas3os2), thereby accelerating wound healing [45, 46]. Moreover, the expression of antimicrobial peptides Cathelicidin and Hepcidin was increased in the PVDF@Fe20-ZIF8 (Janus) group. These antimicrobial peptides not only have direct antibacterial effects but also enhance innate immune responses by activating and regulating immune cells such as neutrophils and macrophages, further eliminating pathogens [47]. The upregulation of antioxidant-related genes, such as Mms19, and a negative regulator of ROS, indicates that the wound dressing can prevent the negative effects of oxidative stress on wound healing. Furthermore, the expression of genes promoting wound healing, such as type III collagen, matrix metalloproteinases (MMPs) MMP-2 and MMP-13, interleukin IL-22, and growth factors Tgfa, Tfgbr3, and Fn1, was also upregulated. Overall, these genes improved wound healing through multiple mechanisms, including antibacterial action, promotion of angiogenesis, cell migration and tissue remodeling, anti-inflammation, and antioxidation, thereby optimizing the wound microenvironment and accelerating the healing process. According to Gene Ontology (GO) enrichment analysis and Kyoto Encyclopedia of Genes and Genomes (KEGG) enrichment analysis (Fig. 6g, h), the results showed that the PVDF@Fe20-ZIF8 (Janus) group exhibited significant enrichment in key biological processes related to wound healing, including inflammatory response, immune response, cell migration, and tissue remodeling compared to the control (-) group. The GO enrichment analysis revealed numerous terms associated with zinc ions, demonstrating their crucial role in the wound dressing for the infected wound healing process. By promoting the transmembrane transport and effective distribution of zinc ions inside and outside the cells, the wound dressing can effectively kill invading bacteria and enhance cellular metabolic activity [48, 49]. Additionally, by supplementing zinc ions, cellular function and repair mechanisms can be optimized, thus providing better wound healing outcomes. Furthermore, the KEGG enrichment analysis results revealed that the PVDF@Fe20-ZIF8 (Janus) wound dressing can regulate inflammation, immune response, cell adhesion, and oxidative stress response through multiple signaling pathways such as HIF-1, PI3K-Akt, and NF-kappa B. Protein interaction network analysis results showed that many genes related to antibacterial activity, such as Gzmg, Serpine1, and Chil3, as well as genes associated with wound healing, like Ccn1 and Tnfaip3, exhibited significant nodal connectivity (Fig. S19). Additionally, gene set enrichment analysis (GSEA) indicated a strong positive correlation in the PVDF@Fe20-ZIF8 (Janus) treatment group for biological processes promoting wound healing, including angiogenesis and extracellular matrix organization (Fig. S20).

Compared with the pure PVDF group, the PVDF@Fe20-ZIF8 (Janus)-treated group exhibited 905 upregulated genes and 742 downregulated genes (Fig. S21). GO enrichment analysis of the PVDF@Fe20-ZIF8 (Janus) group indicated stronger functional enrichment in several critical biological processes (Figs. S22 and S23). The GO terms such as “defense response to bacterium,” “positive regulation of cell activation,” and “positive regulation of vascular wound healing” reflect the enhanced antibacterial properties and improved regulation of cellular behavior. These differences demonstrate the superior antibacterial performance and wound healing promotion of the PVDF@Fe20-ZIF8 (Janus) wound dressing compared to the PVDF membrane. KEGG enrichment analysis indicated that the PVDF@Fe20-ZIF8 (Janus) dressing is enriched in multiple biological pathways related to cellular oxidative stress response, metabolism, proliferation, and immune regulation, such as Toll-like receptor, NF-kappa B, JAK-STAT, Calcium signaling pathways (Fig. S24). These findings suggest that PVDF@Fe20-ZIF8 (Janus) has an enhanced ability to promote cell proliferation and survival, improve immune response, and facilitate cell migration and adhesion, thereby significantly promoting wound healing and improving antibacterial performance. The enrichment in the cytokine–cytokine receptor interaction pathway suggests that PVDF@Fe20-ZIF8 (Janus) enhances the interaction between cytokines and receptors, boosting the responsiveness of the immune system and thus improving antibacterial performance. The enrichment in signaling pathways such as PI3K-Akt and MAPK may significantly enhance cell proliferation and survival capabilities [50, 51]. Moreover, the enrichment in the focal adhesion pathway implies that the dressing improved cell migration and adhesion, aiding the movement of cells from the wound edges to the center, hence promoting wound closure.

Compared with the PVDF@ZIF8 (Janus) group, the PVDF@Fe20-ZIF8 (Janus) group had 542 upregulated genes and 852 downregulated genes (Fig. S25). The GO and KEGG enrichment terms (Figs. S26–S28), such as “zinc ion transport,” “zinc ion import into organelle,” and “glutathione metabolism,” indicate that the PVDF@Fe20-ZIF8 (Janus) wound dressing facilitates the effective uptake and distribution of zinc ions, significantly enhancing cellular utilization of zinc ions, hence improving metabolic activity and immune response capability. Compared to PVDF@ZIF8 (Janus), the PVDF@Fe20-ZIF8 (Janus) dressing shows significant advantages in bacterial defense response, tissue development, and oxidative stress response. Furthermore, the PVDF@Fe20-ZIF8 (Janus) dressing is significantly enriched in several critical KEGG pathways, including the PI3K-Akt signaling pathway, MAPK signaling pathway, cytokine–cytokine receptor interaction, focal adhesion, and extracellular matrix interaction. This indicates that the PVDF@Fe20-ZIF8 (Janus) dressing may enhance the host immune response by regulating the communication and function of immune cells, improving antibacterial performance. Therefore, the PVDF@Fe20-ZIF8 (Janus) dressing promotes cell migration at the wound site, accelerating wound closure and tissue reconstruction.

**Fig. 7:**
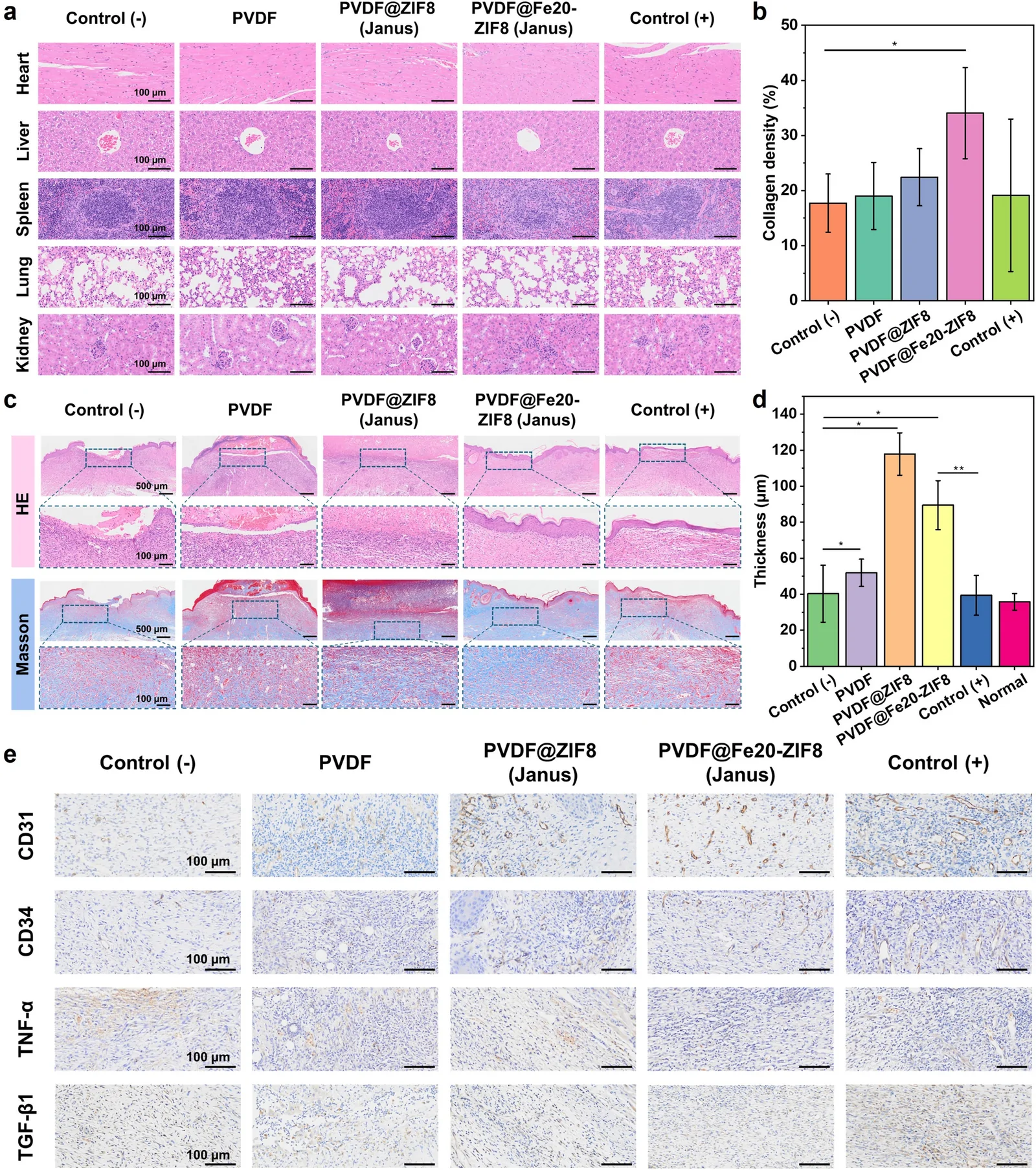
Histological analysis of wound areas using bionic Janus-structured PVDF@FeX-ZIF8 bionic skin. **a** H&E staining of main organs after wound dressing treatment. **b** Quantitative analysis of collagen density across different groups. **c** H&E staining and Masson staining of healed skin area; **d** Quantitative analysis of epidermis thickness. **e** IHC staining of CD31, CD34, TNF-α, and TGF-β1 in various groups. Data are displayed as mean ± SD, *n* = 3. *p < 0.05, **p < 0.01, and ***p < 0.001

Moreover, we analyzed the in vivo biocompatibility of the designed dressings. According to the H&E staining images of the major organs of mice, all organs exhibited normal characteristics, and no significant damage was found, indicating good biocompatibility of the wound dressings (Fig. 7a, b). To evaluate the healing effect of the wound dressings, we conducted H&E and Masson staining on the skin tissues (Fig. 7c). In the PVDF@Fe20-ZIF8 (Janus)-treated group, a continuous epithelial layer and new skin appendages were formed, indicating that wound healing was almost complete. Previous studies have shown that appropriate epidermal thickness can effectively cover the wound, protect newly formed tissue, promote normal wound healing, and prevent excessive extracellular matrix (ECM) deposition and scar formation [52]. Therefore, we measured the epidermal thickness in each group (Figs. 7d and S29). The results showed that the average epidermal thickness in the PVDF@Fe20-ZIF8 (Janus) treatment group (89.50 ± 13.60 μm) was nearly twice that of normal skin (35.82 ± 4.66 μm), which is effective in promoting wound repair [53]. However, other groups had not yet achieved epithelialization, and the average thickness of the formed epidermis was too thin (40.31 ± 15.85 μm) or too thick, which may potentially lead to scar formation (117.90 ± 11.76 μm). Additionally, collagen (mainly types I and III) is a crucial component of newly formed tissue, involved in ECM reconstruction and tissue repair [54]. Quantitative analysis of collagen density can indicate the effectiveness of wound healing (Fig. 7b). The results showed that the collagen distribution in the skin tissue of the PVDF@Fe20-ZIF8 (Janus) group was the most uniform and had the highest density (34.06 ± 8.29%) [55].

In addition, immunohistochemical staining (IHC) was used to quantify angiogenesis and inflammatory cytokine levels during skin tissue repair (Figs. 7e and S30). The results for the commonly used markers CD31 and CD34 in the angiogenesis process showed that the average neovascularization in all treated groups was higher than that in the negative control group (0.65 ± 0.19%), especially in the PVDF@ZIF8 (Janus) group (3.82 ± 0.59%) and the PVDF@Fe20-ZIF8 (Janus) group (4.94 ± 1.81%). The PVDF@Fe20-ZIF8 (Janus) group exhibited the most significant angiogenesis potential. Meanwhile, the negative control group showed the highest levels of the inflammatory cytokine tumor necrosis factor-alpha (TNF-*α*) (9.10 ± 0.89%), while the PVDF@Fe20-ZIF8 (Janus) group showed the lowest levels (1.89 ± 0.68%). In addition, transforming growth factor-beta 1 (TGF-*β*1) acts as an anti-inflammatory agent at various stages of inflammation and wound healing, but excessive TGF-*β*1 can lead to fibrosis. IHC results showed that the PVDF@Fe20-ZIF8 (Janus)-treated group had the lowest TGF-*β*1 expression (5.57 ± 0.70%). However, the positive control group (9.56 ± 0.91%) and the PVDF treatment group (14.17 ± 1.14%) had higher levels than the PVDF@Fe20-ZIF8 (Janus)-treated group. To further explore the transcriptional landscape and gain insights into the potential pathways associated with the wound healing effects of the dressing, we performed qPCR analysis on key markers of angiogenesis and extracellular matrix (ECM) remodeling (Fig. S31). Vascular endothelial growth factor (VEGF) and basic fibroblast growth factor (bFGF) are well-established markers for evaluating angiogenic activity and overall wound repair capacity. Quantitative analysis revealed that, compared with the control and pure PVDF groups, both the PVDF@ZIF8 and PVDF@Fe20-ZIF8 groups were associated with a marked upregulation of these pro-angiogenic factors. Specifically, VEGF expression reached 5.02-fold and 7.01-fold of the control group in the PVDF@ZIF8 and PVDF@Fe20-ZIF-8 groups, respectively, while bFGF expressions were elevated to 1.36-fold and 1.72-fold. In addition, we examined the expression of collagen type I (Col 1) and collagen type III (Col 3). The PVDF@Fe20-ZIF8 group displayed the most pronounced upregulation, suggesting an environment conducive to enhanced collagen synthesis and favorable ECM remodeling. Collectively, this is consistent with our findings in RNA sequence analysis, where the PVDF@Fe20-ZIF-8 group, compared to other groups, exhibited upregulation of angiogenesis-related genes as well as biological behaviors associated with wound healing and tissue remodeling.

## Conclusion

A bionic wound dressing with a Janus structure was designed, fabricated, and investigated, demonstrating that the dressing can effectively promote rapid healing of bacteria-infected wounds. Solvent welding technology, single-sided ZIF modification, and visible light ROS generation mechanism were used to construct the bionic skin wound healing patch. The core factors in the wound management process, such as antibacterial, comfort, protection, and gene expression during wound repair, were thoroughly addressed. By using solvent welding technology, we prepared PVDF@Fe20-ZIF8 with excellent mechanical properties, with a breaking strength of 21.6 MPa and a breaking elongation of 54%, closely resembling human skin. Mechanical simulation confirmed that the welded nanofibers exhibit a uniform internal stress distribution during stretching. Additionally, by controlling nanofiber welding and pore structure, the wound dressing offers good air permeability and high moisture permeability. The Janus structure design enables passive cooling under sunlight and enhances moisture wicking performance in the inner layer, providing a high mid-infrared emissivity and reducing local skin temperature by 4 °C under sunlight. With Fe doping technology, the optimized bionic skin dressing, PVDF@Fe20-ZIF8, exhibits a ROS response to visible light, with twice the ROS response intensity of pristine PVDF@ZIF8, achieving a sterilization efficiency of 97.1%. During the wound healing process, the fabricated bionic skin can regulate gene expression related to wound repair, bacterial defense response, oxidative stress response, and skin tissue development. In summary, the bionic dressing design not only deepens our understanding of wearing comfort but also elucidates the underlying mechanisms of wound repair. Thus, the development of this bionic skin holds promise for advancing wound management and material innovation.

## Supplementary Information

Below is the link to the electronic supplementary material.

Supplementary file1 (DOCX 6805 KB)
