# Supplementary material for: Bionic Cooling Skin for Infected Wound Healing
Source: Nanomicro Lett. 2026 May 28;18:390. doi: 10.1007/s40820-026-02240-6 (PMC13219676; doi:10.1007/s40820-026-02240-6)
Supplement: Supplementary file 1 — Supplementary file1 (DOCX 6805 KB) [file 40820_2026_2240_MOESM1_ESM.docx]

**Bionic Cooling Skin for Infected Wound Healing**

Shuo Shi^1, #^, Huiqun Zhou^2, #^, Yang Ming^1, #^, Xiong Zhou^2^, Hanbai Wu^1^, Haipeng Ren^1^, Lung Chow^2^, Jing Su^3^, Daming Chen^1^, Bin Fei^1^, Joselito M. Razal^1^, Xungai Wang^4*^

^1^ Joint Research Centre for Fiber Innovations and Renewable Materials, School of Fashion and Textiles, The Hong Kong Polytechnic University, 999077, Hong Kong S.A.R, P. R. China

^2^ Department of Biomedical Engineering, City University of Hong Kong, 999077, Hong Kong S.A.R, P. R. China

^3^ College of Textile Science and Engineering, Jiangnan University, Wuxi 214122, P. R. China

^4^ State Key Laboratory of Bio-based Fiber Materials, College of Textile Science and Engineering, Zhejiang Sci-Tech University, Hangzhou 310016, P. R. China

^#^ Shuo Shi, Huiqun Zhou, and Yang Ming contributed equally to this work.

* Corresponding author. E-mail: [xwang@zstu.edu.cn](mailto:xwang@zstu.edu.cn) (Xungai Wang)

**Supplementary Tables and Figures**

**Table S1** Materials and formula for preparing PVDF@Fex-ZIF8

| **Items** | **Fe: Zn** | **FeCl_2_•4H_2_O** | **Zn(NO_3_)_2_•6H_2_O** | **2-Methylimidazole** | **Methanol** | **PVDF** |
| --- | --- | --- | --- | --- | --- | --- |
|  | (mol: mol) | (mg) | (mg) | (g) | (g) | (g) |
| PVD@FZIF8 | 0:100 | 0 | 500.40 | 1.255 | 40 | 0.10 |
| PVDF@Fe5-ZIF8 | 5:95 | 16.79 | 477.62 | 1.255 | 40 | 0.10 |
| PVDF@Fe10-ZIF8 | 10:90 | 33.59 | 452.41 | 1.255 | 40 | 0.10 |
| PVDF@Fe20-ZIF8 | 20:80 | 67.56 | 404.40 | 1.255 | 40 | 0.10 |
| PVDF@Fe30-ZIF8 | 30:70 | 101.38 | 353.85 | 1.255 | 40 | 0.10 |

**Table S2** The primer sequences used for qPCR

| Gene name | Forwards primer | Reverse primer |
| --- | --- | --- |
| VEGF | GCCTCTTCTTCCACCACT | GAAACACGACAAACCCAT |
| bFGF | ACCAGCCTTCCACCCAAA | CGCACCCTATCCCTTCACA |
| Col1 | TTGGGATGGAGGGAGTTTA | TACAGCACGCTTGTGGATG |
| Col3 | ACTGTCAGAGCTACTACGCA | CGGCATCTAACTCTCCGTAG |
| GAPDH | TGTCCCTAATGACAGCTCCTT | GCATCCACCCAAATGACACA |

**Table S3** Surface Element Composition (XPS) of ZIF8 and Fe20-ZIF8

| **XPS atomic % (at.%)** | | | | | |
| --- | --- | --- | --- | --- | --- |
| **Sample** | **C** | **N** | **O** | **Zn** | **Fe** |
| ZIF8 | 61.93 | 24.1 | 6.51 | 7.34 | 0.11 |
| Fe20-ZIF8 | 66.4 | 18.88 | 6.75 | 4.69 | 1.27 |

**Fig. S1** FT‐IR spectra of PVDF electrospun membrane.


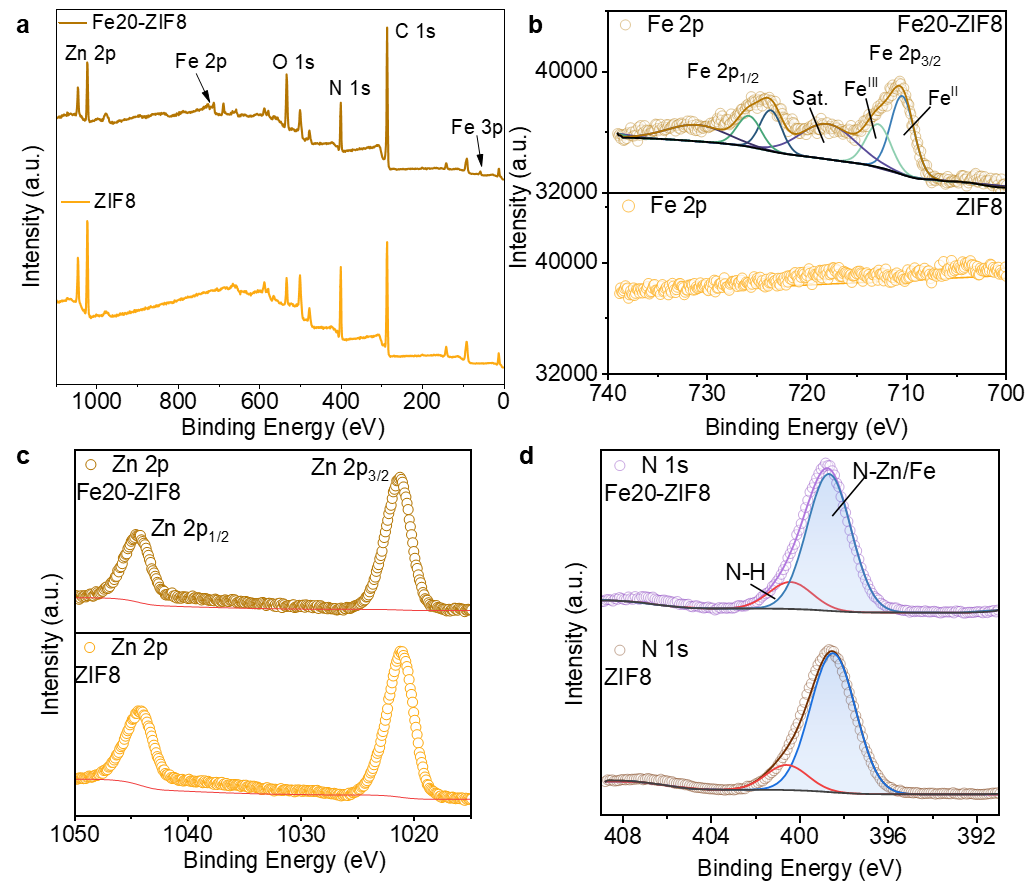


**Fig. S2** (a) X-ray photoelectron spectroscopy survey (XPS), all XPS peaks are calibrated using C 1s peak of 284.8 eV of Fe20-ZIF8 and ZIF8; corresponding (b) Zn 2p, (c) Fe 2p and (d) N 1s high-resolution spectrum.

**Fig. S3** The diameter distribution of PVDF nanofibers.


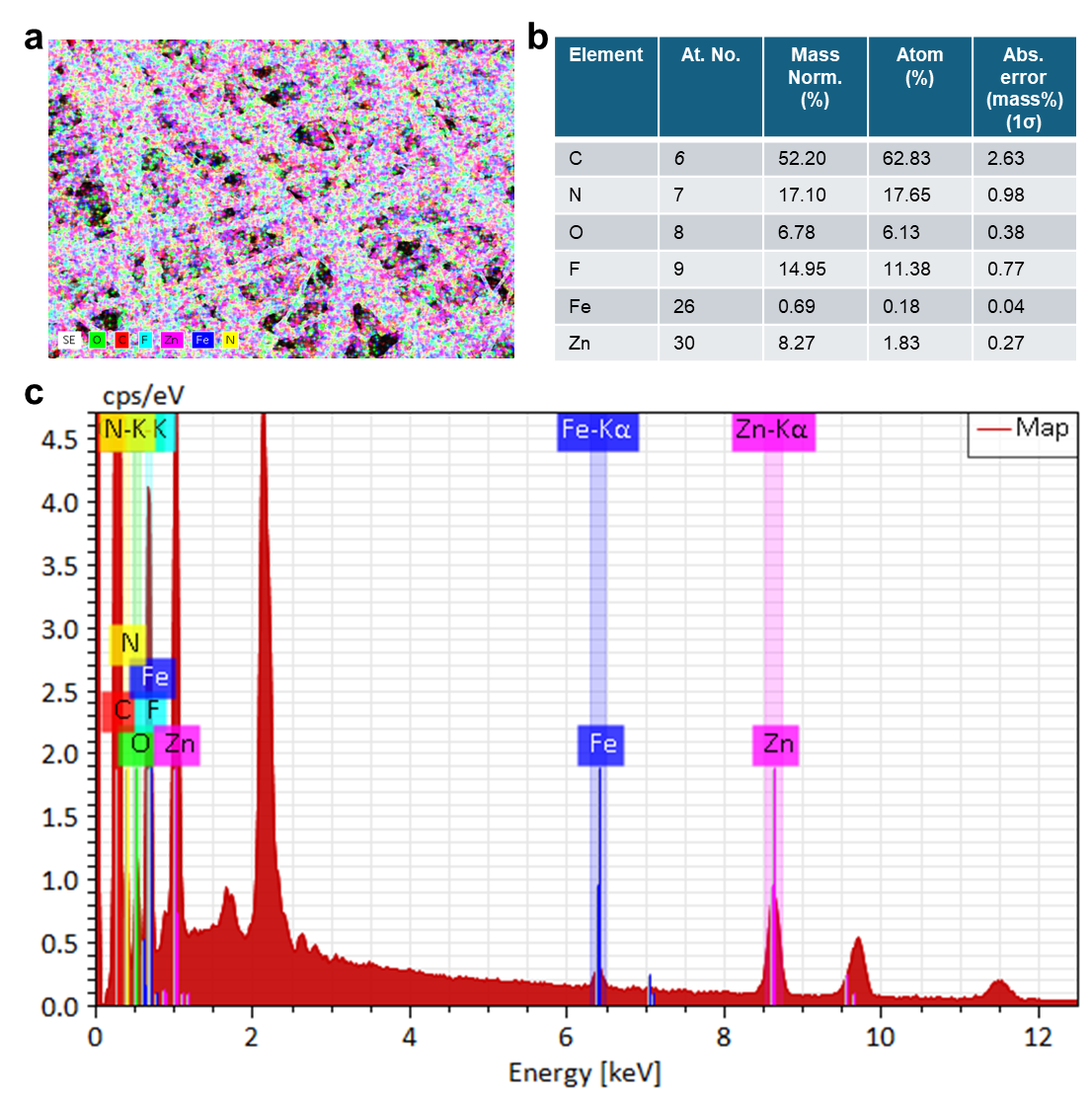


**Fig. S4** EDS elemental mapping image of PVDF@Fe20-ZIF8 electrospun membrane, and atom percentages of the membrane.

**Fig. S5** Band gap of PVDF@Fex-ZIF8 electrospun membrane.


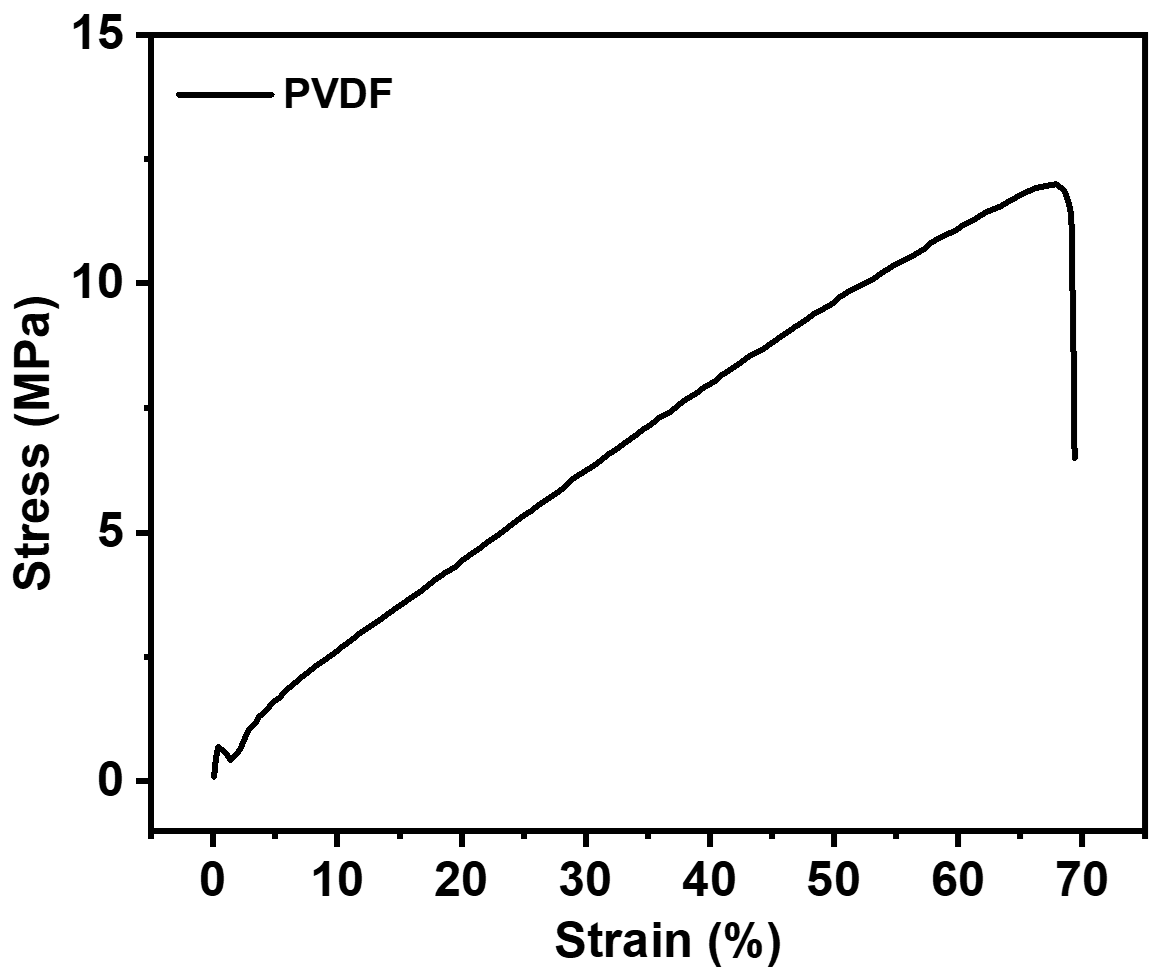


**Fig. S6** Strain-stress curve of PVDF electrospun membrane before treatment.

**Fig. S7** Filtration efficiency of PVDF@Fex-ZIF8 electrospun membrane.


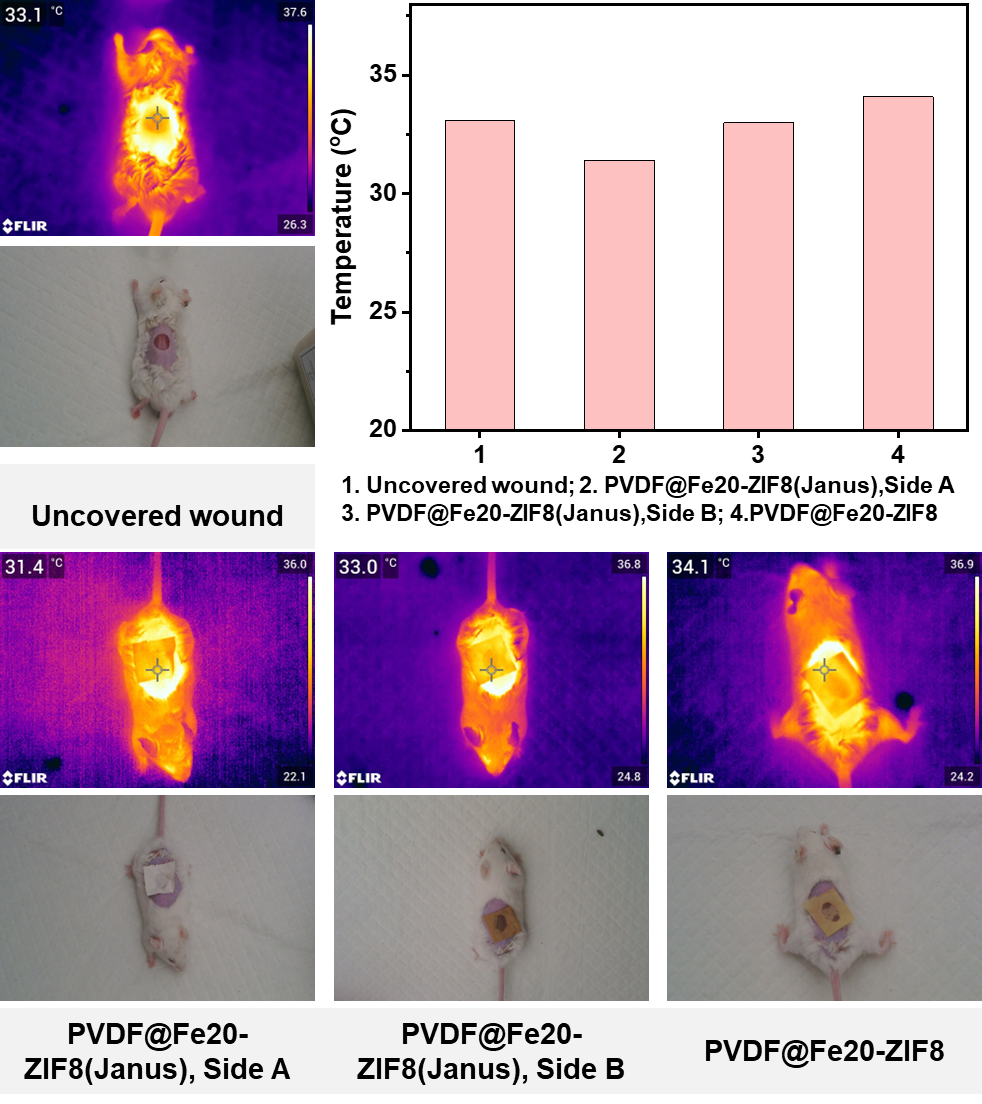


**Fig. S8** Infrared camera pictures of the designed bionic wound dressing, such as PVDF@Fe20-ZIF8, and PVDF@Fe20-ZIF8(Janus) used for wound repair in mice outdoors.


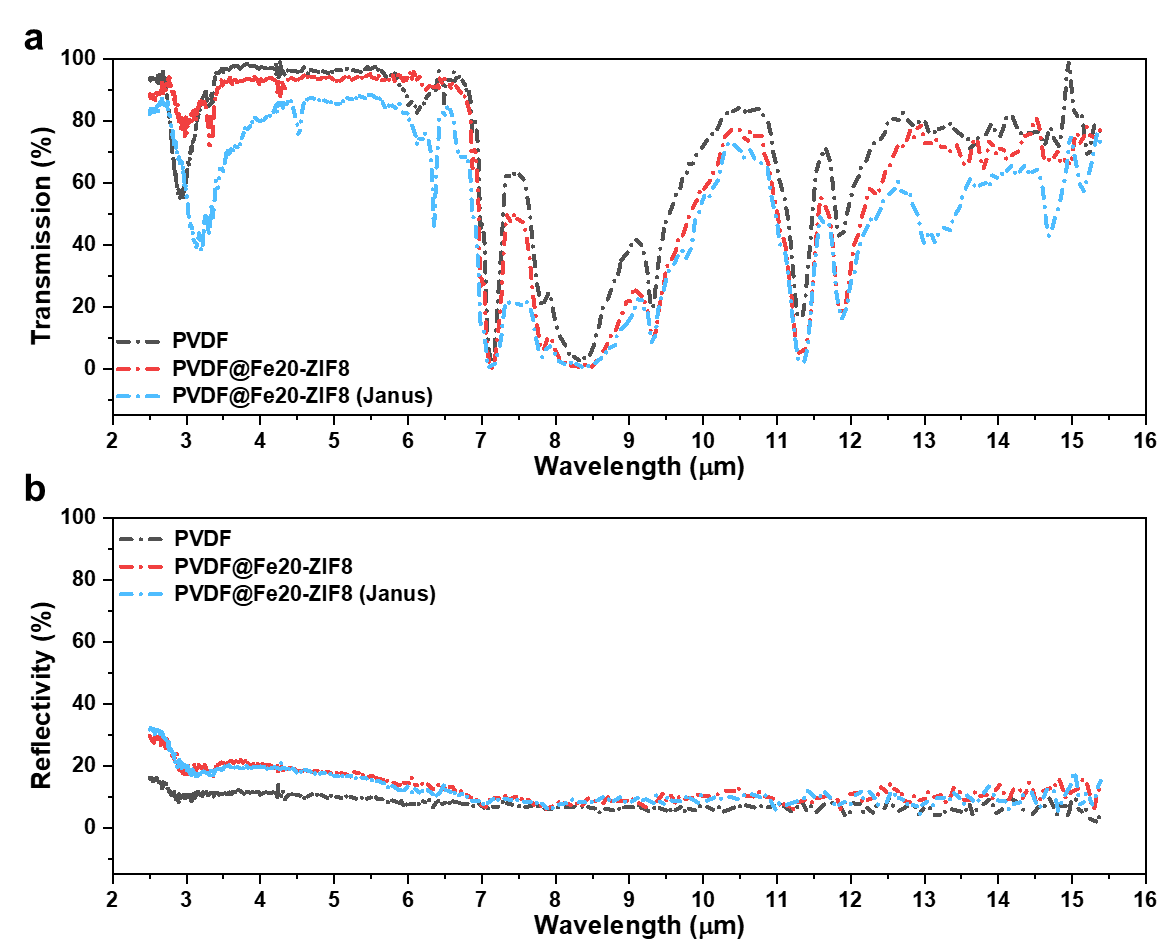


**Fig. S9** Transmission and reflectivity spectra of PVDF, PVDF@ZIF8 (Janus), PVDF@Fe20-ZIF8 (Janus).


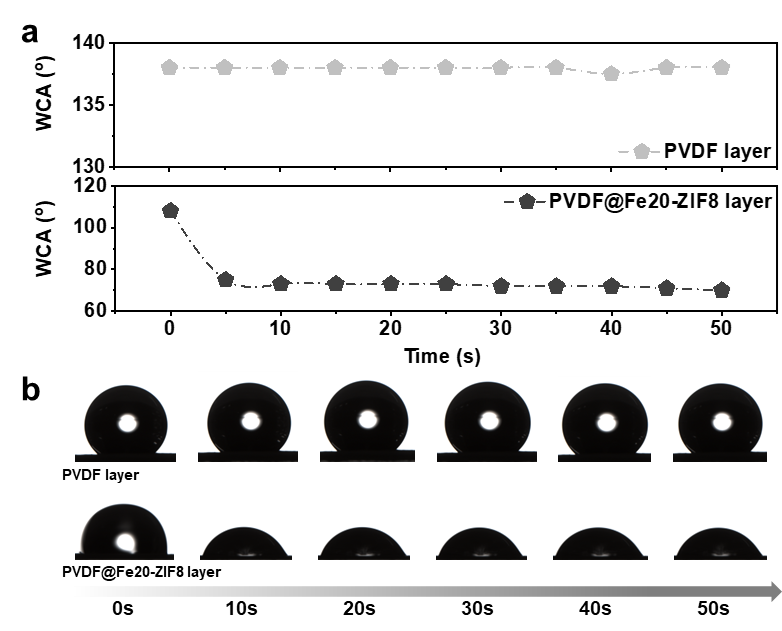


**Fig. S10** The water contact angle of the PVDF layer and PVDF@Fe-ZIF8 layer of the Janus membrane.

**Fig. S11** Body weight of different groups of mice during the wound healing process.

**Fig. S12** White blood cell count, lymphocyte count, monocyte count, and neutrophil count of different groups of mice blood.

**Fig. S13** Platelet count, platelet volume, and platelet distribution of mice blood.

**Fig. S14** Red blood cell count, hemoglobin content, and mean corpuscular volume of mice blood.

**Fig. S15** In vivo antibacterial efficiency of different experimental groups.


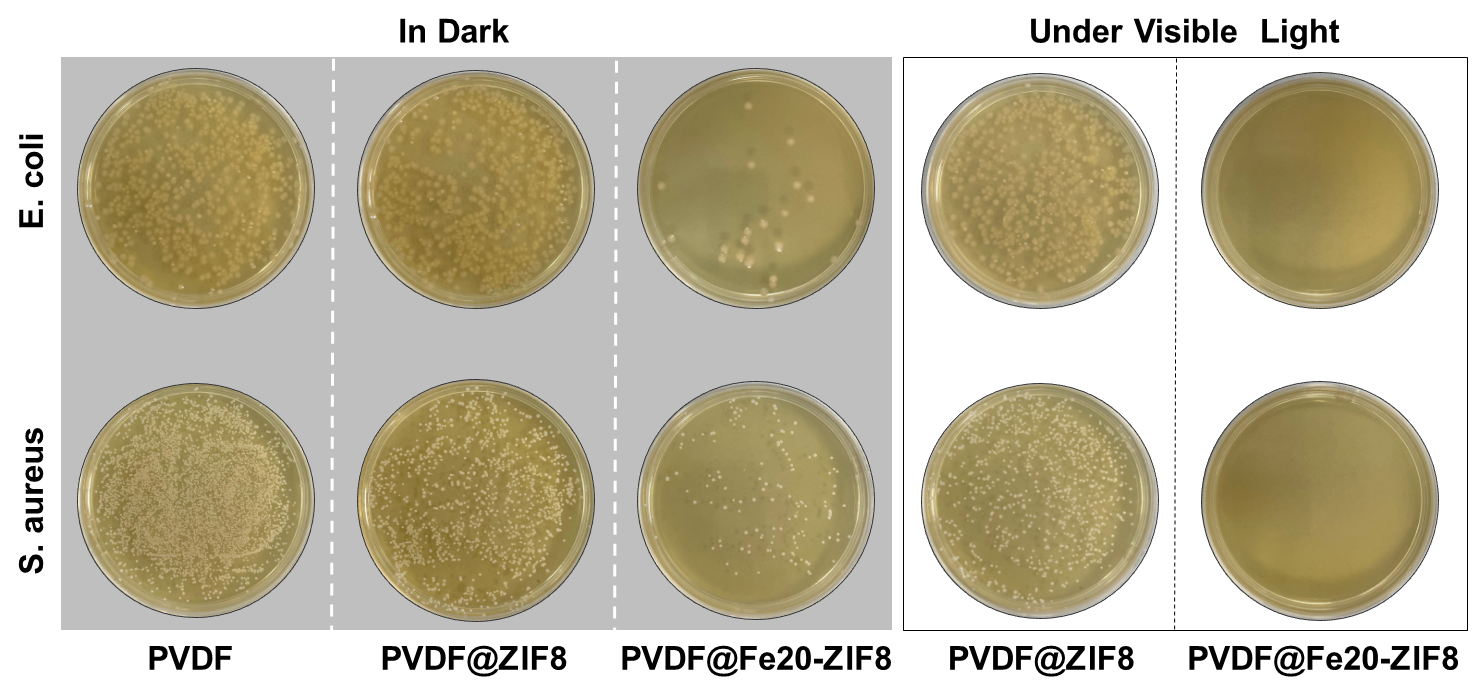


**Fig. S16** In vitro antibacterial assays of PVDF, PVDF@ZIF8, PVDF@Fe20-ZIF8 in dark and under visible light environments.


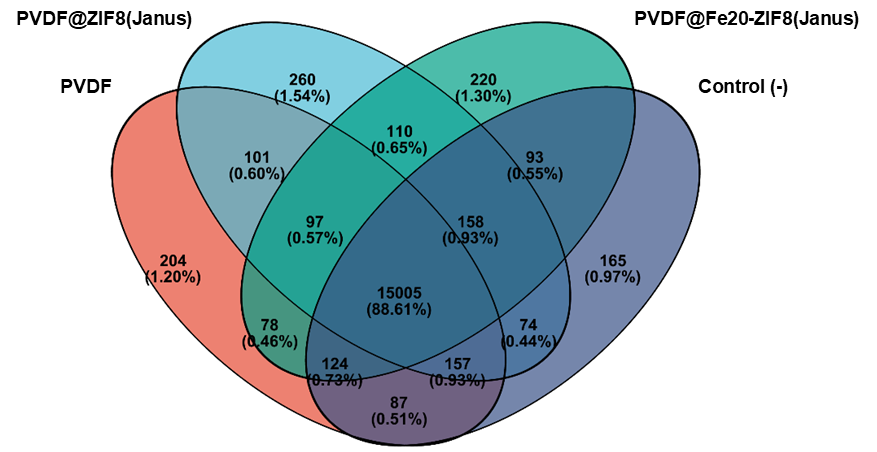


**Fig. S17** Venn analysis of different groups.

**Fig. S18** Differential gene statistics among different groups. S1: PVDF; S2: PVDF@ZIF8; S3: PVDF@ZIF8(Janus); S4: PVDF@Fe20-ZIF8(Janus).


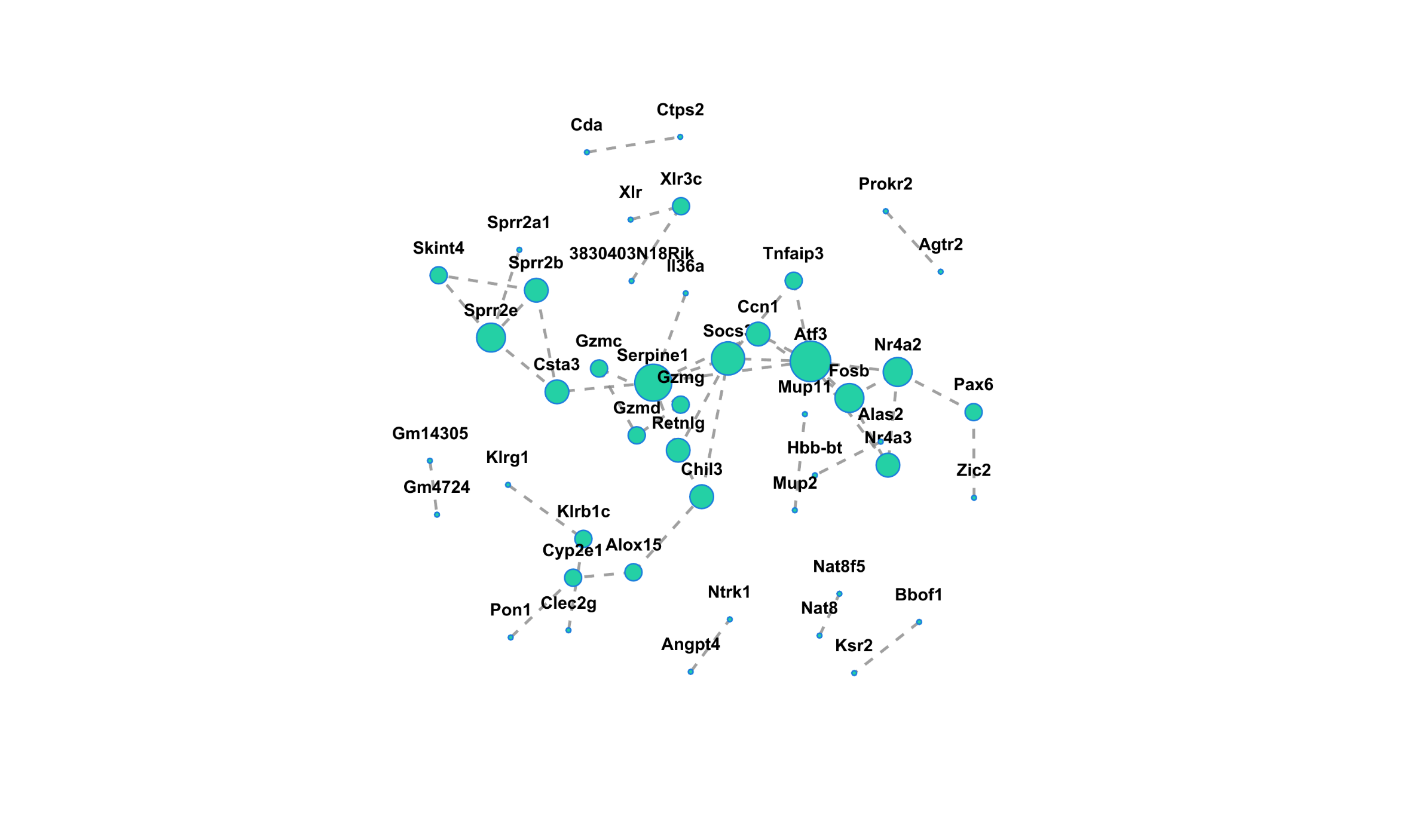


**Fig. S19** Protein interaction network analysis of the genes.


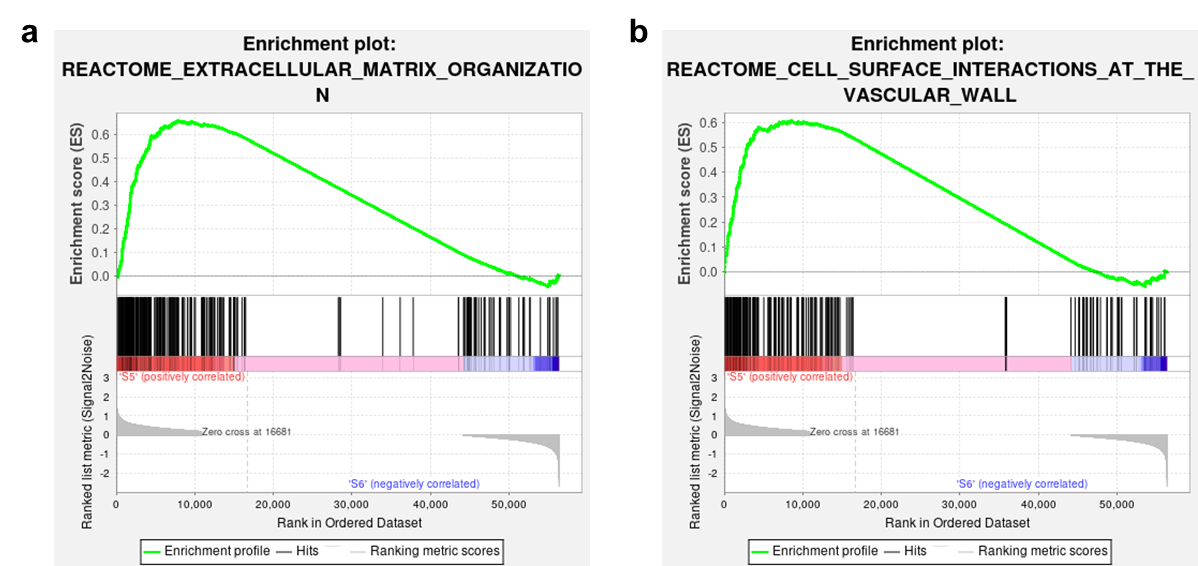


**Fig. S20** GSEA of enrichment situation in the critical gene set.


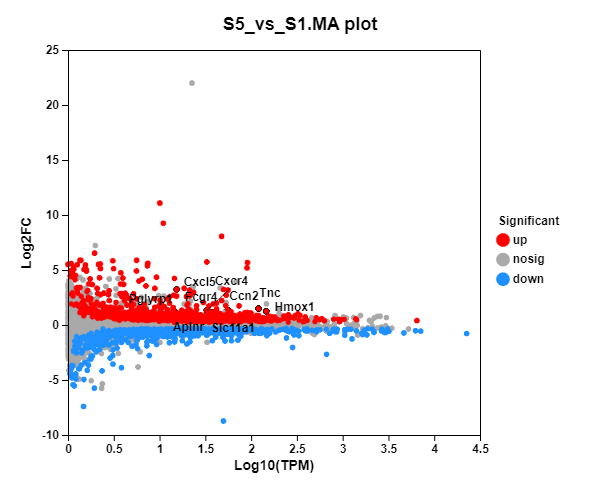


**Fig. S21** MA plot of up and down-regulating genes of PVDF@Fe20-ZIF8 (Janus) group compared with pure PVDF group.


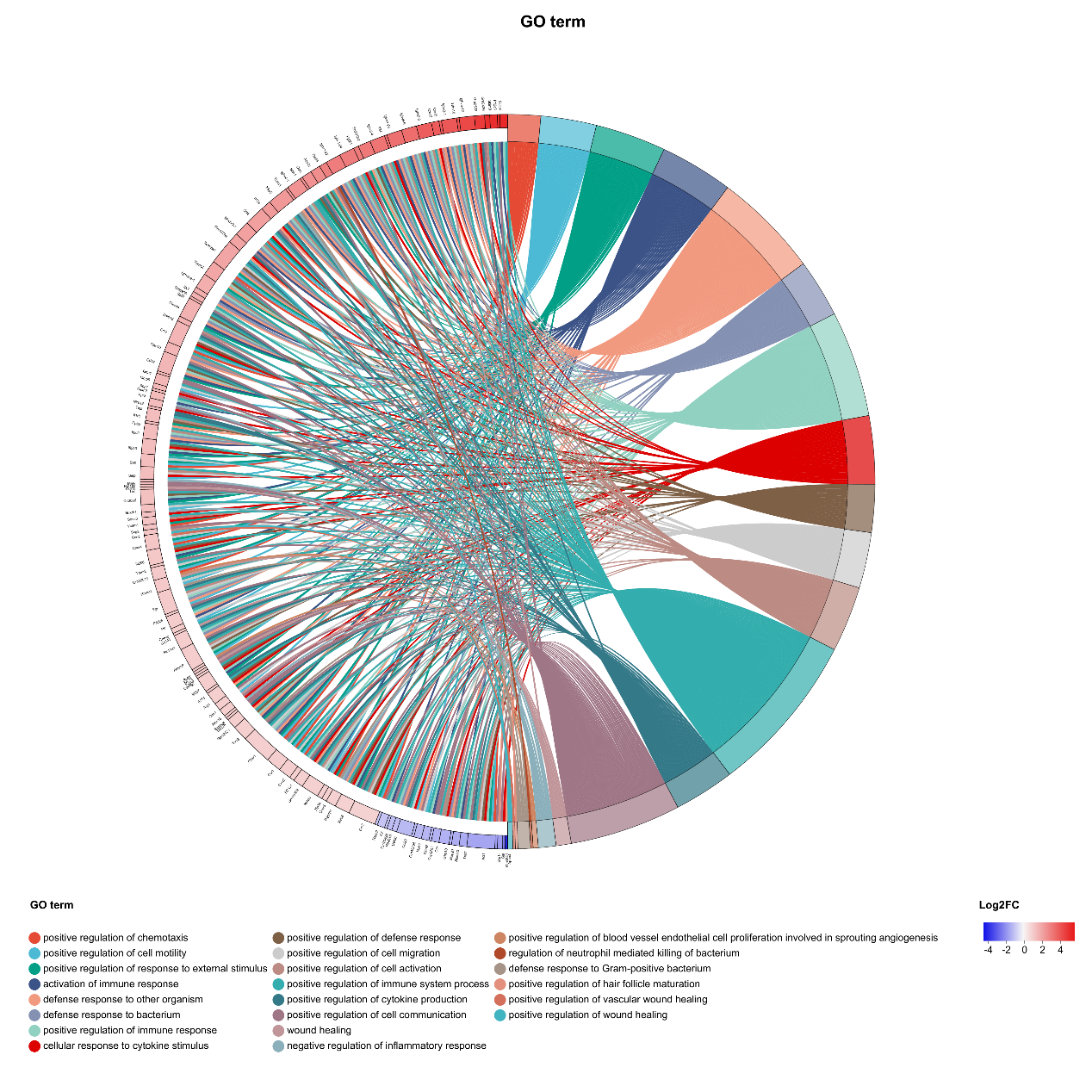


**Fig. S22** GO enrichment analysis of PVDF@Fe20-ZIF8 (Janus) group compared with pure PVDF group with GO term.


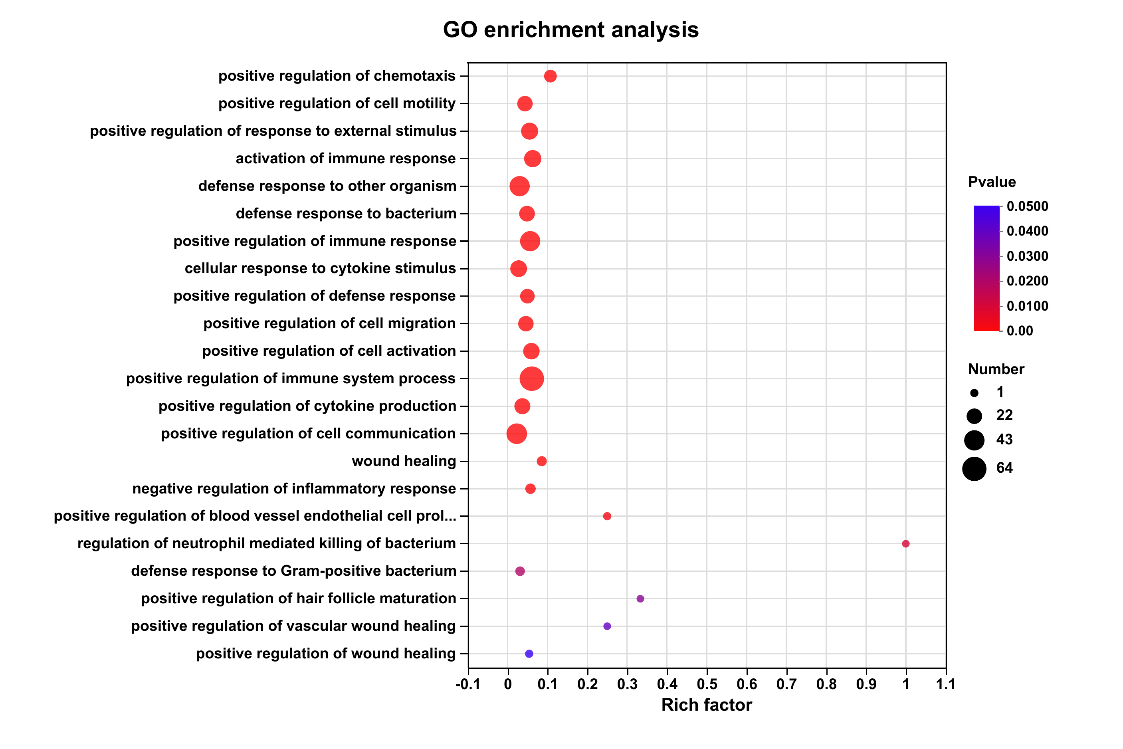


**Fig. S23** Rich factors and the P value of GO term of PVDF@Fe20-ZIF8 (Janus) group compared with pure PVDF group.


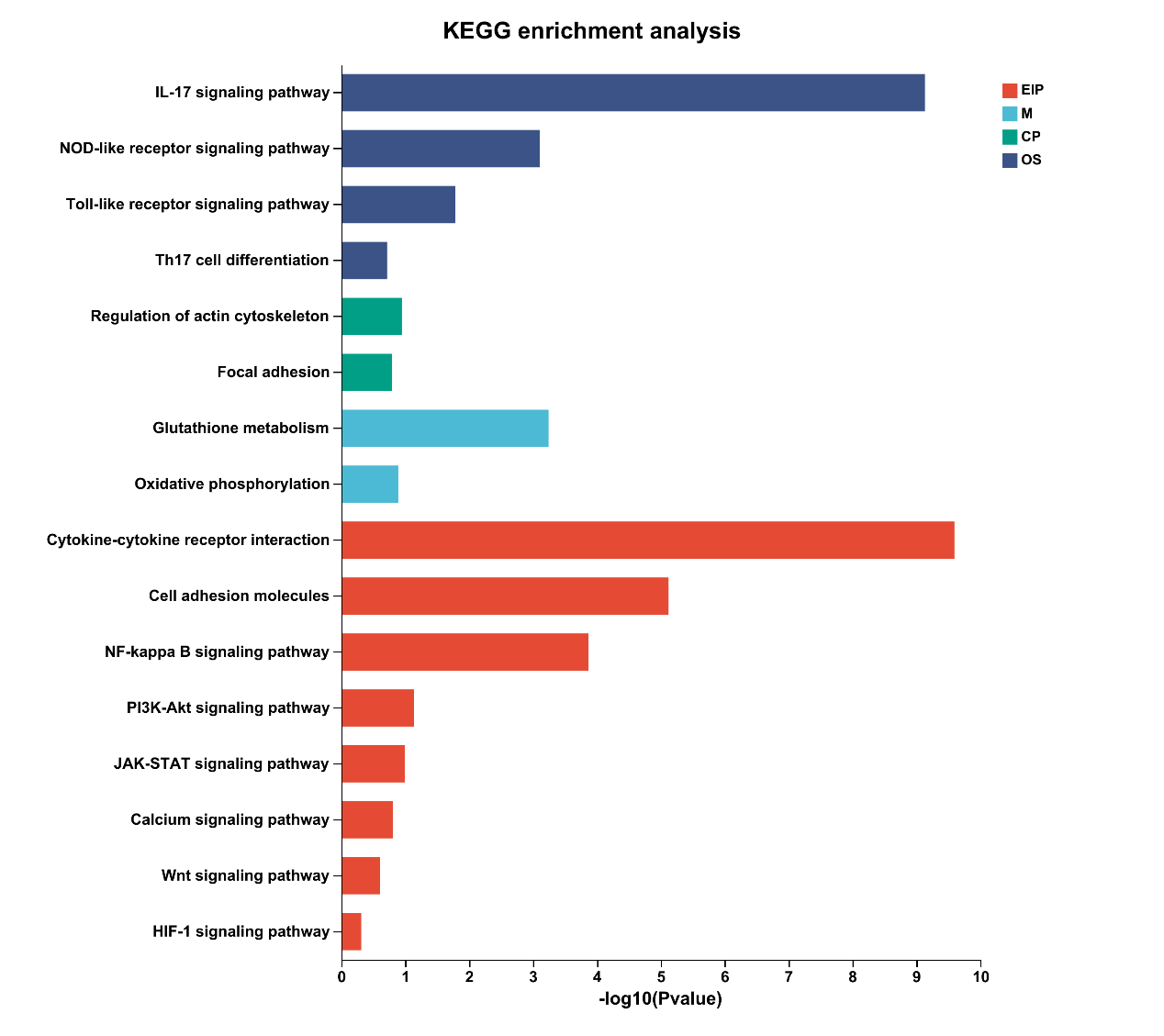


**Fig. S24** KEGG enrichment analysis of PVDF@Fe20-ZIF8 (Janus) group compared with pure PVDF (Janus) group.


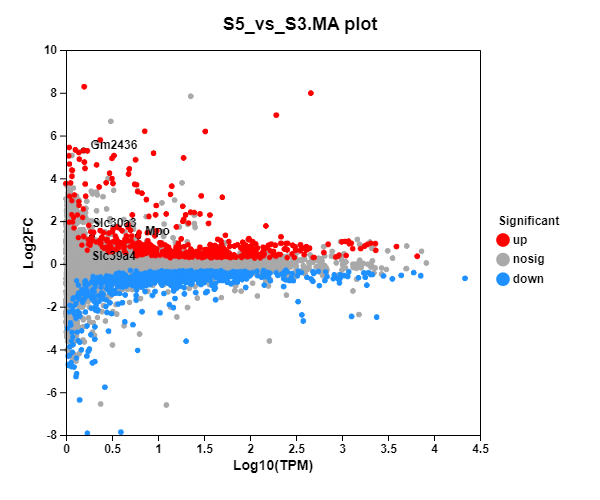


**Fig. S25** MA plot of up and down-regulating genes of PVDF@Fe20-ZIF8 (Janus) group compared with PVDF@ZIF8 (Janus) group.


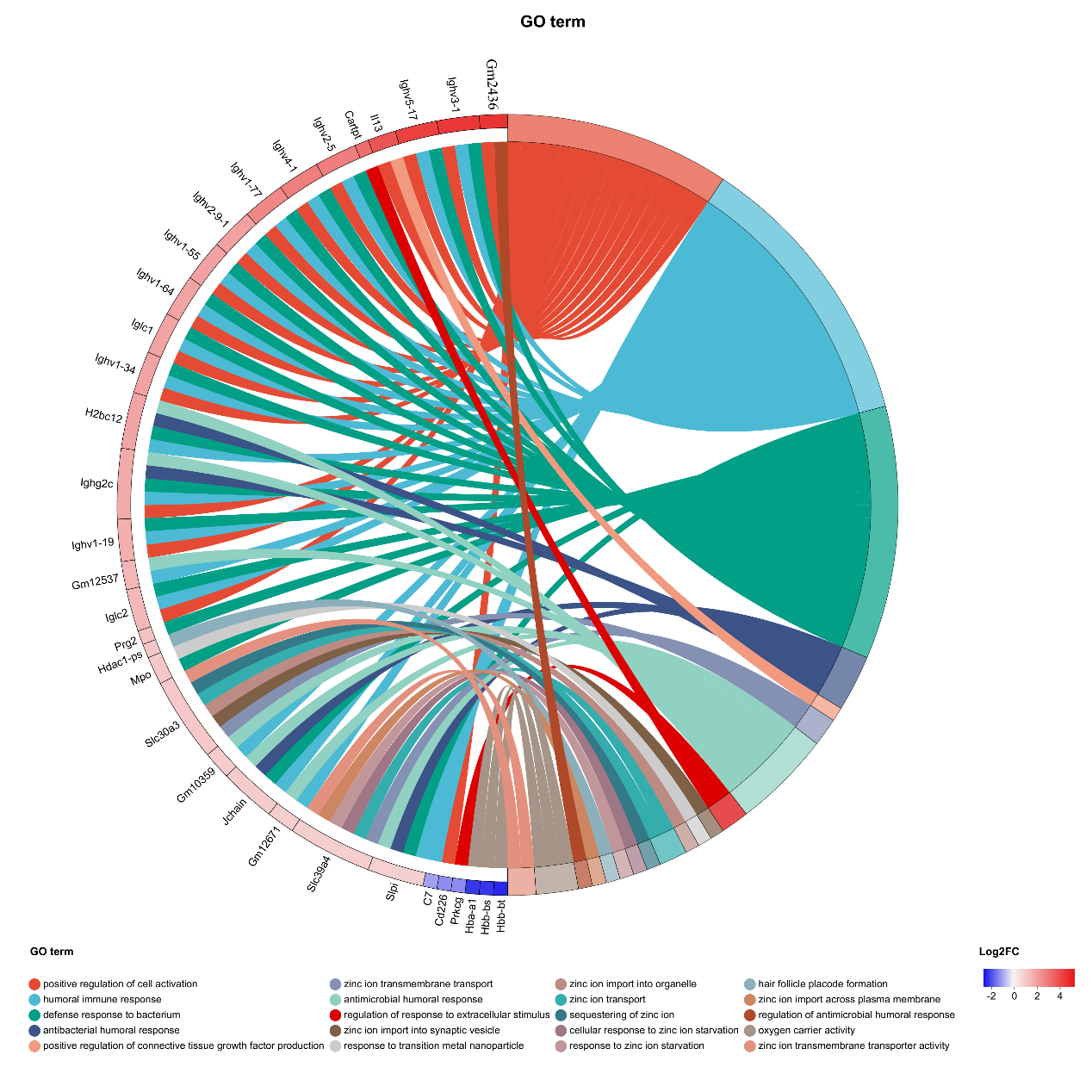


**Fig. S26** GO enrichment analysis of PVDF@Fe20-ZIF8 (Janus) group compared with PVDF@ZIF8 (Janus) group with GO term.


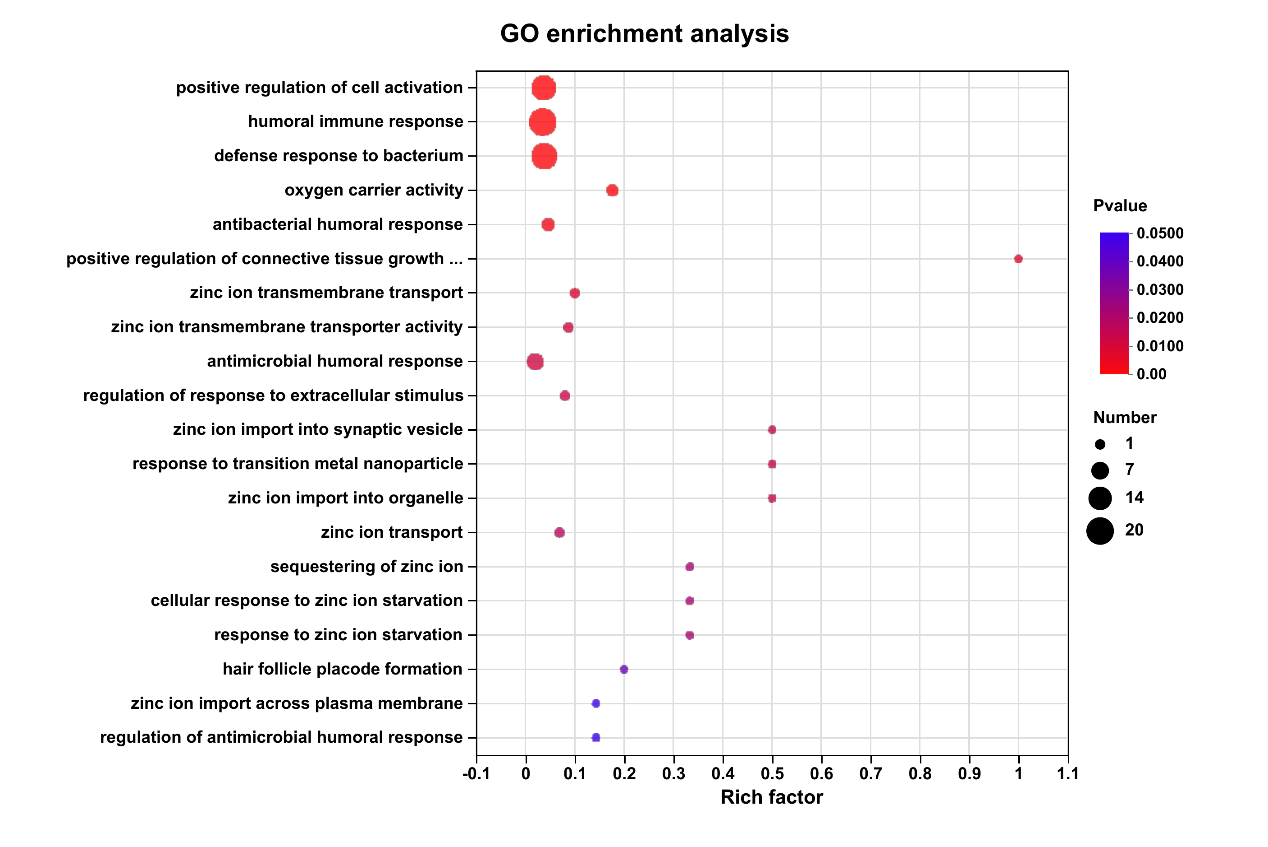


**Fig. S27** Rich factors and P value of GO term of PVDF@Fe20-ZIF8 (Janus) group compared with PVDF@ZIF8 (Janus) group.


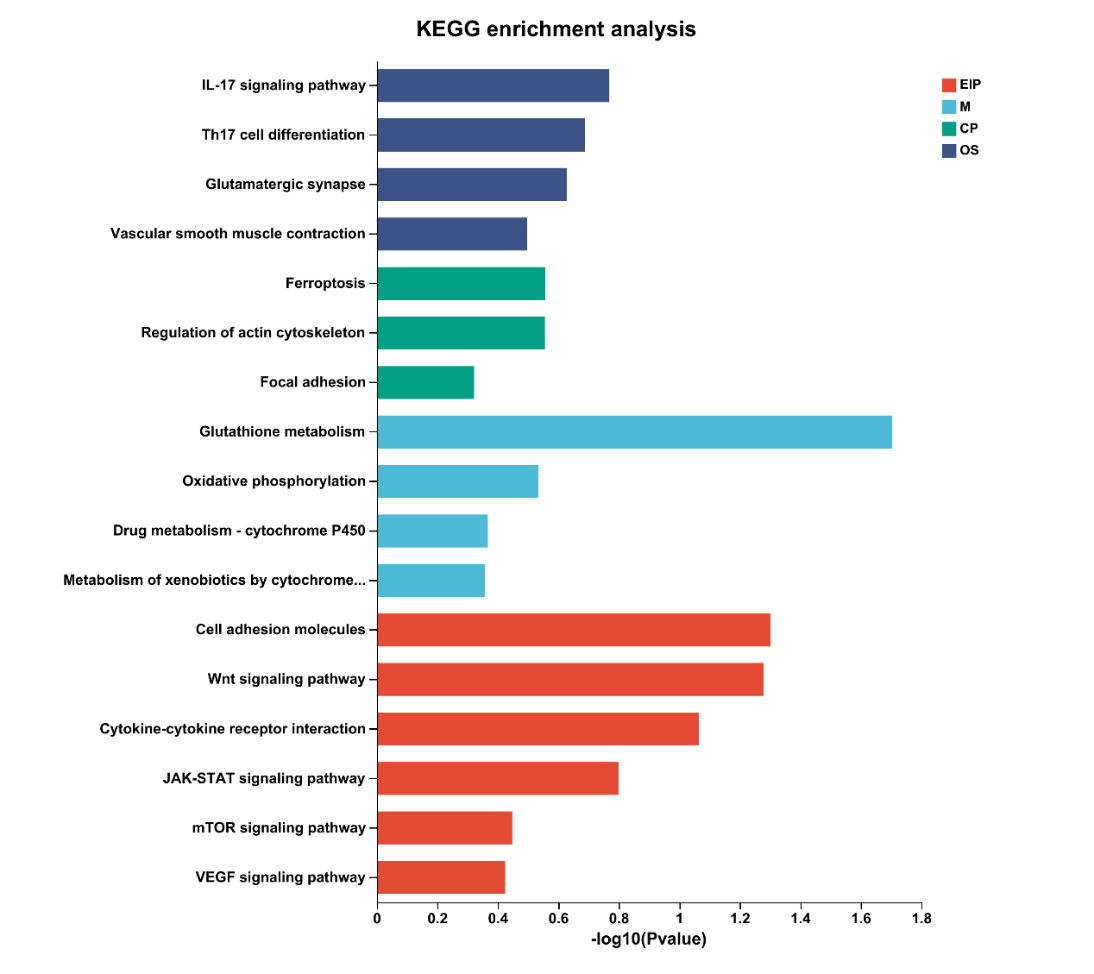


**Fig. S28** KEGG enrichment analysis of PVDF@Fe20-ZIF8 (Janus) group compared with PVDF@ZIF8 (Janus) group.


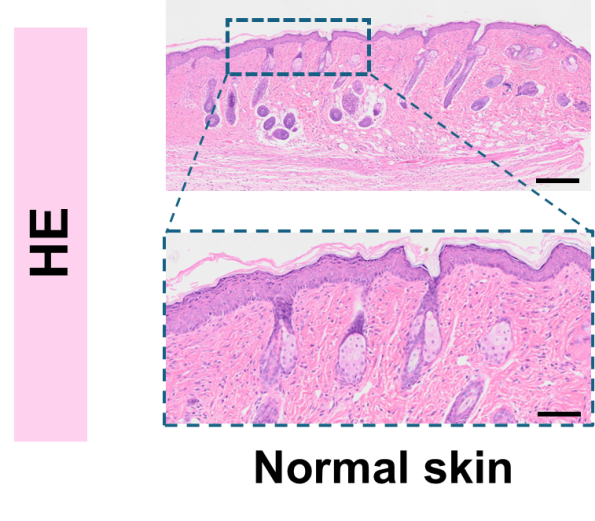


**Fig. S29** H&E staining and Masson staining of normal skin.


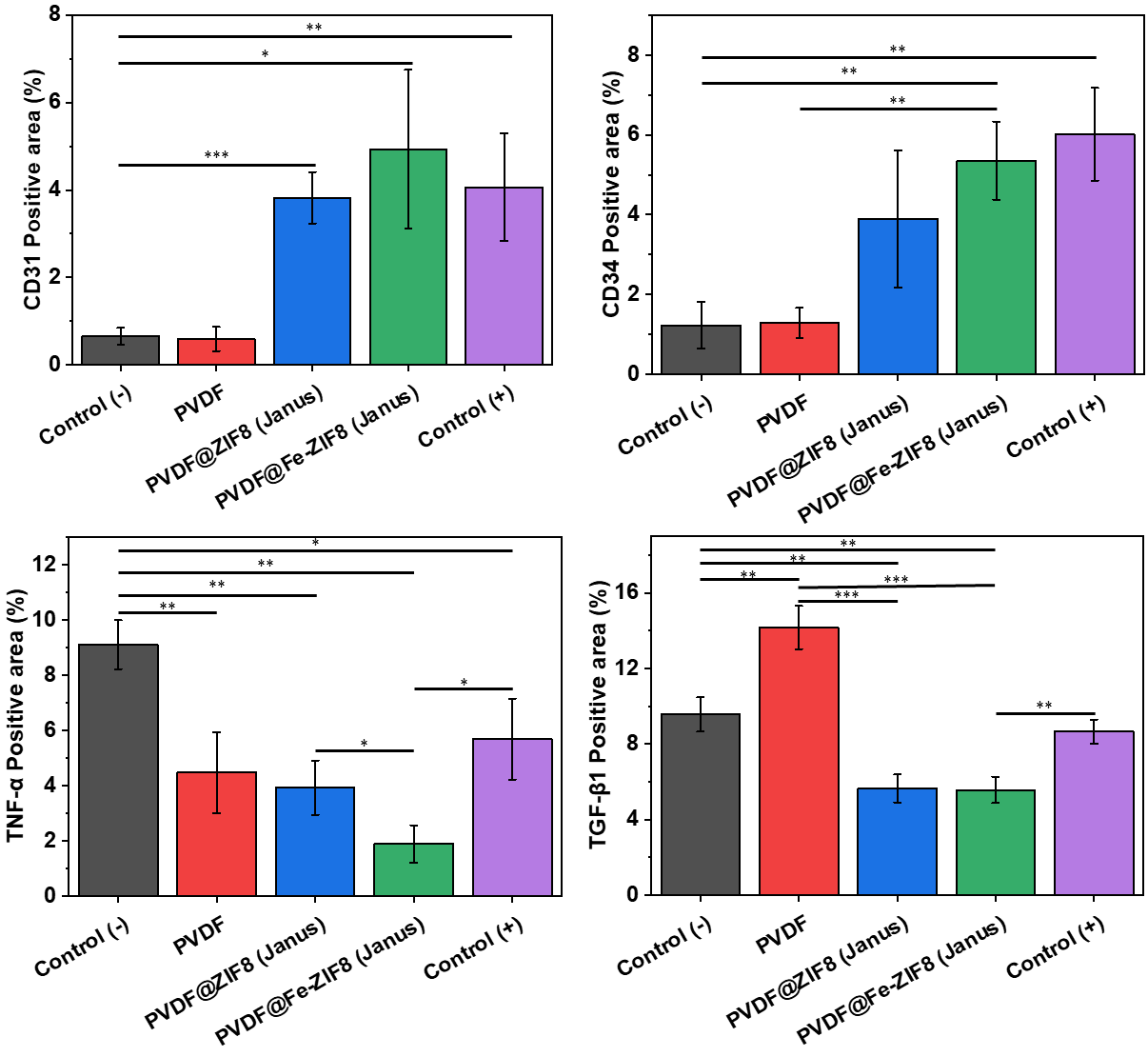


**Fig. S30** Positive area percentages of CD31, CD34, TNF-α, and TGF-β1 in different groups. Data are displayed as mean ± SD, n = 3. *p < 0.05, **p < 0.01, and ***p < 0.001.


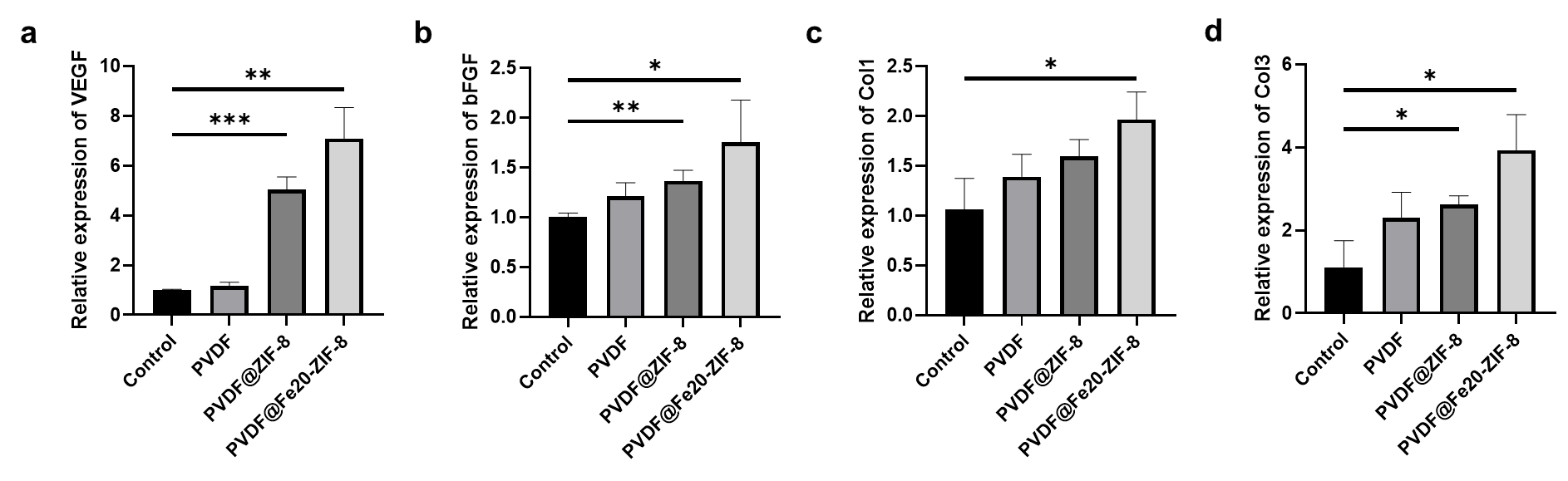


**Fig. S31** PCR analysis of the genes expression related to angiogenesis and wound healing. a) Relative expression of VEGF; b) Relative expression of bFGF; c) Relative expression of Col1; d) Relative expression of Col3. Data are displayed as mean ± SD, n = 3. *p < 0.05, **p < 0.01, and ***p < 0.001.
